# Comparative Investigation of Gene Regulatory Processes Underlying Avian Influenza Viruses in Chicken and Duck

**DOI:** 10.3390/biology11020219

**Published:** 2022-01-29

**Authors:** Selina Klees, Johanna-Sophie Schlüter, Jendrik Schellhorn, Hendrik Bertram, Antje Christine Kurzweg, Faisal Ramzan, Armin Otto Schmitt, Mehmet Gültas

**Affiliations:** 1 Breeding Informatics Group, Department of Animal Sciences, Georg-August University, Margarethe von Wrangell-Weg 7, 37075 Göttingen, Germany; j.schlueter01@stud.uni-goettingen.de (J.-S.S.); hendrik.bertram@stud.uni-goettingen.de (H.B.); antje.kurzweg@uni-hohenheim.de (A.C.K.); faisal.ramzan@uni-goettingen.de (F.R.); armin.schmitt@uni-goettingen.de (A.O.S.); 2 Center for Integrated Breeding Research (CiBreed), Georg-August University, Carl-Sprengel-Weg 1, 37075 Göttingen, Germany; 3 Department of Bioinformatics, Institute for Microbiology and Genetics, Georg-August University, Goldschmidtstr. 1, 37075 Göttingen, Germany; jendrik.schellhorn@uni-goettingen.de; 4 Faculty of Agriculture, South Westphalia University of Applied Sciences, Lübecker Ring 2, 59494 Soest, Germany

**Keywords:** avian influenza, chicken, duck, mallard, gene regulation, differentially expressed genes, RNA sequencing, transcription factor cooperation, master regulators, upstream regulators

## Abstract

**Simple Summary:**

Avian influenza poses a great risk to gallinaceous poultry, while mallard ducks can withstand most virus strains. To date, the mechanisms underlying the susceptibility of chicken and the effective immune response of duck have not been completely understood. In this study, our aim is to investigate the transcriptional gene regulation governing the expression of important avian-influenza-induced genes and to reveal the master regulators stimulating an effective immune response after virus infection in ducks while dysfunctioning in chicken.

**Abstract:**

The avian influenza virus (AIV) mainly affects birds and not only causes animals’ deaths, but also poses a great risk of zoonotically infecting humans. While ducks and wild waterfowl are seen as a natural reservoir for AIVs and can withstand most virus strains, chicken mostly succumb to infection with high pathogenic avian influenza (HPAI). To date, the mechanisms underlying the susceptibility of chicken and the effective immune response of duck have not been completely unraveled. In this study, we investigate the transcriptional gene regulation underlying disease progression in chicken and duck after AIV infection. For this purpose, we use a publicly available RNA-sequencing dataset from chicken and ducks infected with low-pathogenic avian influenza (LPAI) H5N2 and HPAI H5N1 (lung and ileum tissues, 1 and 3 days post-infection). Unlike previous studies, we performed a promoter analysis based on orthologous genes to detect important transcription factors (TFs) and their cooperation, based on which we apply a systems biology approach to identify common and species-specific master regulators. We found master regulators such as EGR1, FOS, and SP1, specifically for chicken and ETS1 and SMAD3/4, specifically for duck, which could be responsible for the duck’s effective and the chicken’s ineffective immune response.

## 1. Introduction

Avian influenza is a viral infection mainly affecting birds such as wild waterfowl or gallinaceous poultry but not stopping at humans or other mammals, and thus posing a high risk for a future pandemic [1]. Its causative pathogen is a type A influenza virus from the *Orthomyxoviridae* family of segmented negative-sense RNA viruses [2]. Based on their pathogenicity in chicken, avian influenza viruses (AIVs) can be classified into high- and low-pathogenic avian influenza viruses (HPAIVs and LPAIVs, respectively) [3]. While chicken can usually withstand an LPAI infection, they succumb to infection with HPAI within a few days. Mallard ducks, on the other hand, are known to successfully fight all LPAI and most HPAI infections, with usually only mild symptoms, and are hence considered a natural reservoir of the virus [1]. After the first report of human infections with HPAI H5N1 in 1997, attention was drawn to the predominantly poultry-affecting avian influenza spreading across the globe [2]. Since 2003, 862 cases of humans infected with H5N1, along with 455 cases of death, were reported to the World Health Organization (WHO) [4].

With the ongoing intensive breeding for different production traits in chicken, such as growth and feed efficiency, other, unanticipated traits such as skeletal defects, metabolic disorders, or immune responses could have been compromised [5]. Therefore, the breeding goals have shifted towards maintaining animal health, leading to both animal welfare and the prevention of economic losses [5].

However, to date, the mechanisms underlying chicken’s susceptibility to avian influenza and the effective immune response of duck have not been completely deciphered. The susceptibility of chicken can be partially explained by their lack of virus pattern recognition receptor RIG-I gene and the gene for the RIG-I binding protein, RNF135, both of which exist in ducks [1,6]. The RIG-I receptor recognizes double-stranded RNA and initiates self-promoting pathways leading to the early type I interferon (IFN) response, which is important for innate immune response. In chicken, other pattern recognition receptors, such as MDA5 and TLR7, are upregulated in response to viral entry, which also leads to the induction of IFN expression [3,6,7,8]. However, the immediate induction of type I IFNs seems to be much more robust and effective in ducks than in chicken or other avian species. In addition to the difference in pattern recognition receptors, there appears to be a variety of factors and differences that lead to the successful or unsuccessful immune response of ducks or chickens, respectively. Different studies evaluated the transcriptomics response to different AIVs in chicken [3,6,9,10,11,12,13,14,15,16,17,18,19,20,21,22], duck [23,24,25,26], or both [1,27,28,29,30]. For example, Smith et al. [1] investigated the role of the expression levels of different interferon-induced transmembrane proteins (IFITMs) in the duck’s ability to alleviate the virus while it prevailed in chicken. Evseev and Magor [7] provide a comprehensive review of the differences in innate immune response in chickens and ducks. However, the host–pathogen interactions and their underlying mechanisms in ducks and chicken are multi-factorial and highly complex, and must be elucidated to obtain a deeper insight into the duck’s effective immune response against AIV while it proves lethal to chicken [7].

Despite the rich literature on the differences in chicken and duck immune response after AIV infection, the role of transcription factors (TFs) and their cooperations, which underlies transcriptional gene regulation, has not yet been extensively studied. The knowledge about the complex interplay of TF pairs could provide promising information to unravel the differences in disease progression in these species, since the TFs specifically bind to the promoter regions of genes and thereby orchestrate differential gene expression in a highly context-specific manner [31,32]. In response to different environmental conditions such as viral infection, they can activate processes or react to specific pathways, and thus fine-tune the gene expression pattern in an organism. By interacting with other TFs in either a cooperative or competitive manner, they form the basis for complex pathway and network structures in biological systems [33,34,35].

To address the limited knowledge about upstream regulators, including TFs, their complex interplay, and master regulators, which are responsible for an effective immune response after avian influenza infection, we performed a systematic analysis using an RNA-seq dataset. More specifically, mainly considering the effective immune response of duck, we identified the corresponding differentially expressed genes (DEGs) in response to the virus and analyzed their promoter regions to determine the upstream regulators. Then, to investigate the regulatory mechanisms of these DEGs in chicken, we analyzed their chicken orthologs to assess the species-specific regulators. Focusing on the master regulators arising from enriched TFs and TF-TF cooperations, our results can help to resolve the question of why the relevant genes could be differentially expressed in duck, while transcriptional gene regulation in chicken remains unsuccessful. Consequently, in our results, we present two groups of master regulators for ileum and lung: while the first group of master regulators contains common regulators found for both species, the species-specific master regulators were assigned to the second group. In particular, we strive to decipher the duck-specific master regulators related to the immune responses that are absent in chicken. Our findings could be essential in the search for possible mechanisms that stimulate an effective immune response in ducks while dysfunctioning in chicken.

## 2. Materials and Methods

**Figure 1:**
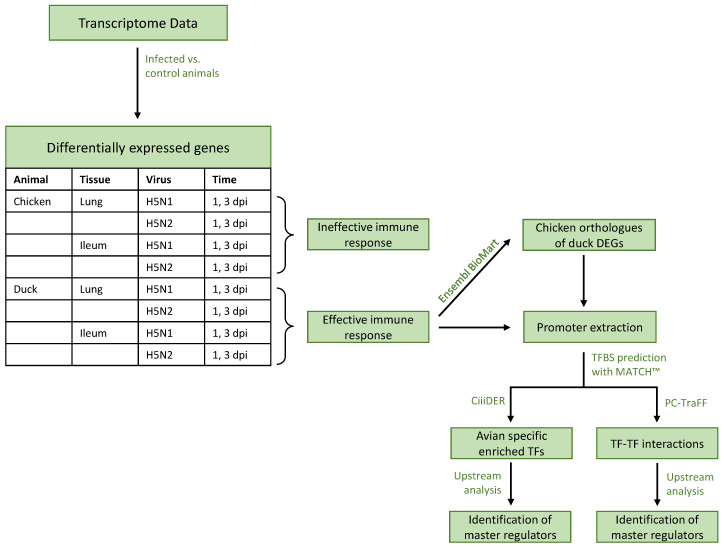
Flow chart of the employed analyses. Differentially expressed genes (DEGs) were derived by comparing the gene expression rate of a specific condition against a mock infection for that condition (e.g., chicken lung at 1 dpi with H5N1 infection against chicken lung at 1 dpi with mock infection). TF and TFBS stand for transcription factor and transcription factor binding site, respectively. H5N1 is a high-pathogenic avian influenza virus (HPAIV), while H5N2 is a low-pathogenic avian influenza virus (LPAIV).

In this section, we describe the methods, starting at the transcriptome level where differentially expressed genes are identified. Since the avian-influenza-induced differential expression of genes in duck have been abundantly compared to chicken, and duck is generally known to effectively prevent severe disease progression, we have a particular interest in investigating the promoter regions of DEGs in duck that potentially allow duck to adapt to the H5N1 virus and enable a proper immune response, which is apparently not the case for the orthologous genes in chicken. Thus, we want to identify the diversity in gene expression by applying promoter analyses to duck and chicken and identify the transcription factors that may provide an explanation for their varying immune responses. An overview of the steps encompassed in our analysis is given in Figure 1.

### 2.1. Transcriptome Data

The RNA-sequencing analysis of lung and ileum tissue samples from chickens and ducks infected with high- (H5N1) and low- (H5N2) pathogenic avian influenza viruses measured 1 and 3 days post-infection (dpi) was conducted by Smith et al. [1]. In their study, a total of 20 white leghorn chickens and 20 Domestic Gray Mallards were challenged with either the HPAI or the LPAI virus. Processed RNA-sequencing data, e.g., count tables for the mapped reads and experimental design, were retrieved from Array Express under the publicly available accessions *E-MTAB-2908* and *E-MTAB-2909* for chicken and duck, respectively. For each experimental condition (e.g., chicken, lung, H5N1 infection, 1 dpi), gene expression was measured for three biological replicates, resulting in a total of 24 samples from infected animals and 12 mock-infected control samples for each species. In chicken and duck, the expression of 24,356 and 25,952 genes was measured, respectively. For further details on the experimental design, as well as the processing steps of the RNA-sequencing data, we refer to the study by Smith et al. [1].

The identification of DEGs was performed in R by using the state-of-the-art package DESeq2 (version 1.30.0) [36] with default parameters for the median-of-ratios normalization and the ashr R package (version 2.2-47) for log${}_{2}$ fold change (LFC) shrinkage [37]. DEGs were determined for each condition (e.g., lung infected with H5N1 at 1 dpi) against a control group (e.g., lung with mock infection at 1 dpi). Similar to the study of Smith et al. [1], genes were considered to be significantly differentially expressed if the criteria |LFC| > 0.58 and the FDR-adjusted *p* value < 0.05 were met.

### 2.2. Identification of Enriched TFs and TF-TF Cooperations

To unravel the differences in transcriptional gene regulation underlying the identified DEGs, we focused on their regulatory regions (promoter regions) and identified enriched TFs as well as TF-TF cooperations using the two bioinformatics tools CiiiDER [38] and PC-TraFF [35,39], respectively. A detailed description of the theory behind both methods can be found in the original studies [35,38,39]. Besides some algorithm-specific parameters, both algorithms require as input the promoter sequences and a library of position weight matrices (PWMs) representing the TFBSs.

- **Promoter sequences:** Using the current versions of reference genomes GRCg6a and CAU_duck1.0, we extracted the promoter sequences ranging from −1000 base pairs (bp) to +100 bp relative to the transcription start site (TSS), similar to previous studies [35,40,41,42]. Sequences were rejected if the full promoter sequence could not be obtained, which was mostly the case for genes on scaffolds.
- **Creation of the PWM profile and TFBS detection:** Following our previous studies [31,32], we created a custom avian-specific PWM profile. For this, we first downloaded the TFs of avian species (chicken, duck, turkey, zebra finch, and flycatcher) from animalTFDB 3.0 [43] and selected those that were expressed in at least one RNA-Seq experimental condition. Second, we mapped the TFs to the PWMs stored in the TRANSFAC database (release 2018.1) [44]. Finally, we clustered the PWMs hierarchically based on their pairwise Pearson’s correlation coefficients and selected the representative with the highest information content for each cluster in order to create a non-redundant PWM profile with thresholds minimizing the sum of the false-positive and false-negative rates (“minSUM profile”). In total, the profile contains 553 PWMs, which are provided in File S1. We predicted the transcription factor binding sites by applying the MATCH${}^{TM}$ tool [45], which obtains the custom avian-specific PWM profile and a matrix library provided by TRANSFAC [44] as input.
- **TF enrichment:** We performed a TFBS enrichment analysis by employing the CiiiDER tool [38] in order to identify over- and underrepresented TFBSs. In the following, we refer to a TF as over-/underrepresented in a condition if its corresponding TFBS is significantly over-/underrepresented in the set of promoter sequences of the respective DEGs compared to a custom background. The background set is composed of the promoter sequences of those genes that were not differentially expressed in any of the conditions. From this, the custom background was created as a subset of sequences of the same global GC distribution as the foreground sequences using BiasAway [46]. In a last step, a random sample of equal size was taken as the foreground gene set from the custom background for each gene set, which eventually led to individual background sets from the same distribution, thus making them comparable. Assessment of the distributions of TFBS predictions in foreground and background promoter sets is carried out by an FDR-adjusted p value threshold of 0.05.
- **TF-TF Cooperation:** The PC-TraFF algorithm [35] and its extension PC-TraFF+ [39] are well-established, information-theory-based approaches to identify TF-TF cooperation pairs using the concept of pointwise mutual information. While PC-TraFF detects the co-occurring TFBSs of TF-pairs in the promoter sequences, PC-TraFF+ separates the highly sequence-set-specific TF-cooperations from the common ones by removing the background co-occurrences of TFBSs. The algorithm needs the predefined distance thresholds as input for the TFBSs. As in our previous studies [31,32], we used the recommended distances of ≥5 and ≤20 and defined a TF-pair as significant if its z-score ≥ 2.

### 2.3. Identification of Master Regulators

Similar to previous studies [40,47,48,49,50,51], we detected upstream regulators that regulate a set of DEGs through concerted coordination of TFs and intermediary modulators. More precisely, these so-called master regulators (key nodes) are found on top of the regulatory hierarchy of complex regulatory networks, leading to the finely tuned gene expression of a gene set. In order to identify master regulators targeting the TFs and their partners, we applied the so-called “upstream analysis” provided by the geneXplain platform, which is based on a modified shortest-path algorithm [40,51,52]. Consequently, focusing mainly on H5N1, we established the top five master regulators for the lung and ileum tissues of chicken and duck using the GeneWays database [53].

### 2.4. Annotations and Ortholog Mapping

The orthologs were retrieved from the BioMart web services [54] via the R package biomaRt [55]. It is important to note that the mapping of, e.g., duck DEGs to chicken orthologs is not necessarily bijective, since a duck gene could be missing in chicken (e.g., *RIG-I*), and thus have no chicken ortholog, or a duck gene could have two orthologs in chicken.

## 3. Results and Discussion

**Figure 2:**
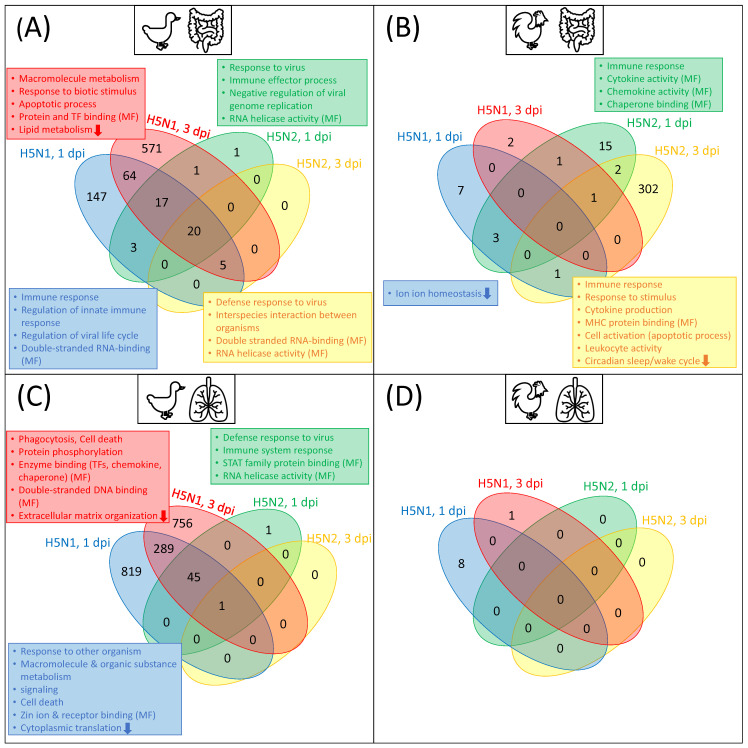
Venn diagrams of the DEGs (**A**) duck in ileum, (**B**) chicken in ileum, (**C**) duck in lung, and (**D**) chicken in lung with selected enriched Gene Ontology (GO) terms. The DEGs are obtained by comparing animals infected with AIV (H5N1 (HPAI) or H5N2 (LPAI)) with mock-infected animals. The colors within the venn diagram, as well as the colors of the GO-term boxes, stand for the respective condition: blue represents H5N1 infection 1 dpi, red represents H5N1 infection 3 dpi, green represents H5N2 infection 1 dpi, and yellow represents H5N2 infection 3 dpi for each species and tissue. Within the boxes, an arrow down indicates that the GO-term is enriched among the downregulated DEGs; otherwise, the terms are enriched among the upregulated DEGs. The GO-terms represent biological processes except, if stated differently, in the form of MF (molecular function). Venn diagrams are based on the data provided in Table S1.

In this study, by analyzing a transcriptome dataset, we firstly identified differentially expressed genes (DEGs) for lung and ileum tissues in chicken and duck after infection with H5N1 and H5N2 at 1 and 3 dpi. In line with the results of Smith et al. [1], our analysis of RNA-Seq data with DESeq2 revealed three different observations: (i) we detected a considerably higher number of DEGs in the duck than in chicken under most conditions (see Table 1 and Table S1); (ii) the vast majority of DEGs were highly context-specific with regards to the virus strain and timepoint. Only 20 and 1 were found to be common in all conditions in the duck ileum and lung, respectively, while no DEG was observed for all conditions in chicken (see Figure 2); (iii) the response in terms of differential expression was higher after infection with the HPAI H5N1 compared to infection with the LPAI H5N2, epspecially in duck (see Table 1). The gene set enrichment analysis of the DEG sets based on Gene Ontology (GO) classification demonstrates that differential gene regulation after virus infection deviates between chicken and duck (Figure 2). The full lists and treemaps for GO enrichment are given in Table S2 and Figures S1 and S2.

To summarize, in agreement with previous studies [1,27,28,29,30], the DEG analysis indicates that the general pattern of differential gene expression differs greatly between duck and chicken after AIV infection. In particular, the infection with H5N1 elicits a rapid and effective immune response in ducks, whereas the chicken immune system did not appear to respond to the same extent.

Despite the great interest in and rich research on avian influenza, there is still a lack of knowledge about the underlying transcription factors and their combinatorial interplay orchestrating gene expression and leading to an effective immune response in ducks while failing in chicken. In order to reveal transcriptional gene regulation factors that play important roles in disease progression, we compared the upstream regulatory regions (i.e., promoters) of the duck DEGs with those of the respective chicken orthologs. Since the response regarding differential expression appears to be most pronounced after infection with the H5N1 virus—and, as an HPAIV, poses the greatest risk for avian as well as mammal species—we concentrate on this virus in the following.

Typically, in bioinformatics, the choice of the threshold value for, e.g., FDR-adjusted *p*-values, is of great importance for the number of significant results. In this study, we mainly followed the values used in the study of Smith et al. [1] to ensure some comparability. Nevertheless, it is important to note that a *p*-value may be interpreted differently in different species, e.g., due to a lower variability of transcriptomics data in genetically stable inbred lines, such as the chickens used in this study, compared to ducks. A more stringent *p*-value threshold of 0.01 for the DEG identification or TFBS enrichment analysis leads to a strong reduction in their results, which, in turn, results in an insufficient number of genes or TFs for further analysis (for a *p*-value comparison, see Tables S1 and S3). For this reason, we used a threshold of 0.05 in the following analysis.

Several studies have investigated the importance of glycosylation with respect to viral entry and replication [56,57,58]. Glycosylation is a post-translational process of host cells that can be used by AIVs to attach glycan moieties to their own proteins [56]. In our DEG sets, we observed one enriched GO term related to glycosylation (GO:MF glycosaminoglycan binding). Remarkably, this GO term was enriched among both up- (duck, lung, H5N1, 1 dpi) and downregulated (duck, lung, H5N1, 3 dpi and duck, ileum, H5N1, 3 dpi) DEG sets, but its interpretation is beyond the scope of this study.

### 3.1. Transcription Factor Binding Site Enrichment

**Figure 3:**
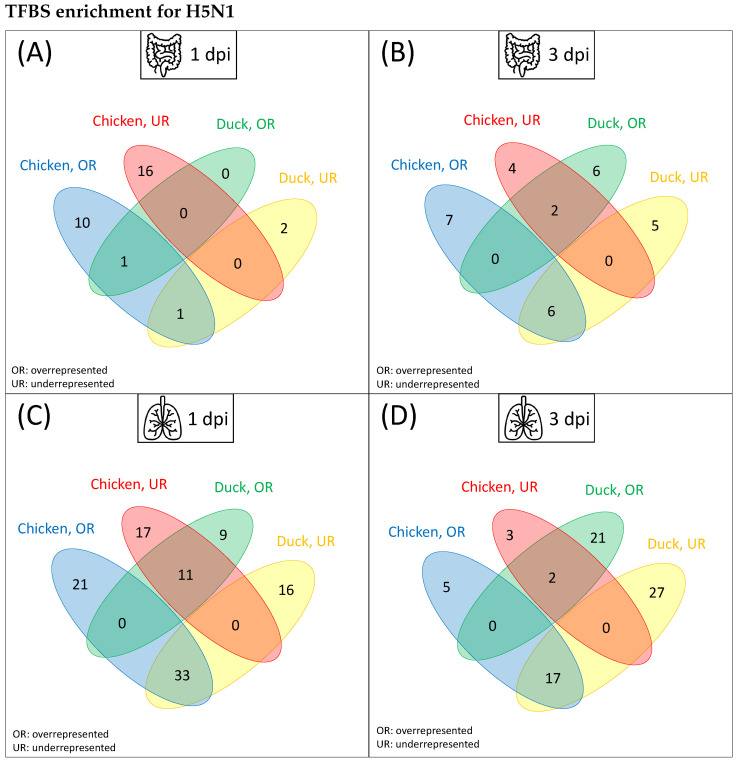
Venn diagrams of TFBS enrichment to compare over- (OR) and underrepresented (UR) binding sites in chicken and duck. The promoter regions of DEGs (after infection with HPAIV H5N1) in duck and the corresponding orthologous genes in chicken were extracted to obtain the over- and underrepresented TFBSs. (**A**) shows the corresponding number of TFBSs for the ileum 1 dpi, (**B**) shows the ileum 3 dpi, (**C**) shows the lung 1 dpi and (**D**) shows the lung 3 dpi. Venn diagrams are based on the data provided in Table S3.

In a first step, we identified significantly over- or underrepresented TFBSs in the promoter regions in the gene sets. The Venn diagrams of over- and underrepresented TFBSs in duck and chicken show a similar pattern for both tissues and timepoints: a high number of enriched TFBSs are unique to either chicken or duck, resulting in only a slight overlap between chicken and duck in terms of over- or underrepresented TFBSs (Figure 3). Interestingly, when comparing overrepresented TFBSs in duck and underrepresented TFBSs in chicken or vice versa, there appears to be more overlap. Generally, the number of predicted TFBSs that are significantly over- or underrepresented in the ileum is smaller in both chicken and duck than in lung, which reflects the corresponding numbers of DEGs. To offer a closer insight into the related TFs of the enriched TFBSs found for the H5N1 infection, we explain their functions in more detail. As the HPAIV is known to predominantly replicate in the respiratory tract [1], we will further concentrate on the lung tissue with functional interpretation. The lists of significantly over- or underrepresented TFBSs are provided in Table S3.

Based on the enriched TFBSs in chicken at 1 dpi, we observed 21 TFs that were uniquely overrepresented in chicken and 33 TFs that were overrepresented in the chicken promoters while underrepresented in the duck promoters (Figure 3). We observed many TFs of the basic helix–loop–helix (bHLH) class and the C2H2 zinc finger class, including different TF families, such as zinc finger proteins (ZNFs), Zinc finger and BTB domain-containing proteins (ZBTB), or specificity proteins (SPs). Furthermore, TF families such as SMAD, AP2, TFII-I, GCM, and paired box factors (PAX) can be found [59]. Similar TF families are salient after 3 dpi in chicken, with a greater focus on zinc finger factors, as they make up 13 out of 22 chicken TFs. For both timepoints, we observed several tryptophan cluster factors, including a TF from the interferon regulatory factor family (IRF4) and ETS/ETS-related TFs.

Interferon regulatory factors (IRFs) play a major role in the immune response by inducing several processes and pathways upon avian influenza infection. For example, the over-expression of IRF7 in chicken DF-1 cells resulted in a higher viral replication and cell death rate than in control cells upon infection with LPAI H6N2 [9]. Transcriptome analysis revealed that chicken IRF7 could be involved in the modulation of programmed cell death via pathways such as the TGF-β, FOXO, and the JAK-STAT pathway [9].

Interestingly, binding sites of the SMAD family members SMAD4 and -5 were enriched in chicken at both timepoints, but not in duck promoters. The SMAD factor family is tightly linked to the TGF-β pathway, which is involved in various immune-related processes such as apoptosis, the innate immune response by type I interferon production, or early pulmonary fibrosis via epithelial–mesenchymal transition in response to influenza A virus (IAV) infection [9,60,61,62,63]. As a response to IAV invasion, the RIG-I-like receptor (RLR) signaling, followed by IRF3 activation, represses TGF-β-induced SMAD signaling in mammal cells [62]. Hence, the availability of SMAD binding sites could be an important regulator of TGF-β and RLR signaling in chicken.

The ETS/ETS-related TF family is uniquely enriched in the chicken promoters. Apart from various cellular processes ranging from embryonic development to apoptosis and carcinogenesis, ETS factors play a role in both the innate and adaptive immune response [64]. Interestingly, it has recently been shown that the ETS-family member ETV7 targets several interferon-stimulated genes (ISGs) to negatively regulate the effective IFN-mediated control of influenza viruses, and can thus be considered as a suppressor of the type I IFN response in mammalian cells [65]. Hence, an over-representation of different ETS binding sites in chicken promoters could possibly influence the intensity of the antiviral type I IFN response, which should be investigated in future studies.

In the duck lung at 1 dpi, 9 TFs are uniquely overrepresented (Figure 3). Among them, we found representatives of the TF families forkhead box (FOX) (FOXC1, FOXL2, FOXO3, and HNF3B), POU (POU3F2 and TST1), STAT, homeobox (HOX), and one IRF TF (IRF4). Another 11 TFs, which were also overrepresented in the duck promoter sets, were simultaneously underrepresented in chicken. Here, we predominantly found homeo domain factors such as HOXD13, NKX22, NKX61-62, DLX3, LHX3, PRX2, and SIX3. The pattern of significantly enriched TFs in duck 3 dpi is similar to that of 1 dpi. One TF family that is more prominent 3 dpi is the HOX family and we further observed the C2H2 zinc finger factor SALL3 while the IRF4 disappeared at 3 dpi.

The FOX family of TFs is suggested to be involved in the regulation of a variety of processes, such as cell growth, proliferation, differentiation, longevity, immunology, and cell-cycle control [66]. FOX TFs play an important role in the FOXO signaling pathway, which regulates important processes such as stress resistance, cellular proliferation, and apoptosis [9,67]. The subclass FOXO is known to be involved in the regulation of lifespan and diseases by orchestrating processes such as cell-cycle progression and apoptosis under severe stress conditions in mammals, and FOXO was shown to be a negative regulator of IRF7, a member of the interferon regulatory factor family [9,67].

Interestingly, the binding sites for two members of the signal transducer and activator of transcription (STAT) family, a main factor of the JAK-STAT signaling pathway, are enriched at both timepoints in the duck lung, but not in chicken. This highlights the importance of the JAK-STAT pathway, which is one of the key pathways in type I IFN response and induces interferon-stimulated genes (ISGs) [9,68,69]. In particular, virus entry followed by IFN expression leads to an IFN receptor-associated Janus-kinase (JAK) phosphorylation, which activates STAT TFs to enhance target IGS gene expression [70,71]. Hence, a lack of enriched binding sites for STAT factors in the chicken promoter sequences could possibly result in a weaker upregulation of ISGs and less efficacy in the JAK-STAT pathway.

Another TF family whose binding sites are overrepresented only in the duck promoters is the POU family. Interestingly, there is evidence that members of the POU family, expressed in B and T cells, may interact with STAT3 and can activate different interleukin promoters, which are related to immune and inflammatory responses in human cells [72].

Additionally, the genes of some promising enriched TFs, e.g., IRF7 (*ENSAPLG00000012752*), STAT1 (*ENSAPLG00000013226*), and STAT4 (*ENSAPLG00000023296*) are significantly upregulated upon AIV infection in the duck lung 3 dpi, which may underline their importance in response to the virus.

### 3.2. TF-TF Cooperations

**Figure 4:**
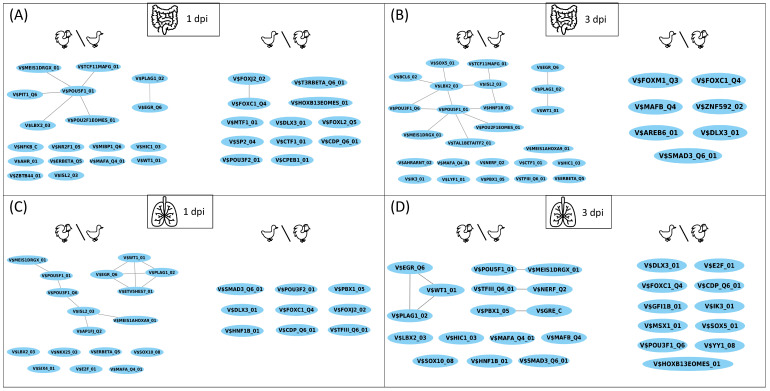
Differences in transcription factor (TF) cooperation networks found by the PC-TraFF algorithm for (**A**) ileum 1 dpi, (**B**) ileum 3 dpi, (**C**) lung 1 dpi and (**D**) lung 3 dpi with HPAIV H5N1. The difference in nodes in the networks of duck and chicken is denoted by the set difference sign (∖). The nodes are labeled by the PWM names representing TFs, as given by TRANSFAC [44]. They follow the structure V$*factorname_version*, where “V$” indicates that the PWM originates from a vertebrate TF, *factorname* specifies the name of the corresponding TF, and *version* is specified to uniquely identify the PWM. The networks were visualized with Cytoscape [74]. Full size image is provided in Figure S3.

To obtain a closer insight into the disease regulation progress in chicken and duck, knowledge of the complex interplay between TFs could provide further essential information, since they are important for the regulation of the transcriptional machinery and form the backbone for the fine-tuned adaptation of a species to specific environmental conditions [35,39]. By further focusing on the HPAIV, we applied the PC-TraFF algorithm [39] and identified the cooperation of TFs based on their binding site co-occurrence patterns in the promoter regions of the investigated genes in the two species. Based on the PC-TraFF results, we constructed a TF cooperation network, in which the nodes represent the TFs and the edges indicate their cooperation. The complete networks for lung and ileum are provided in Table S4 and Supplementary File S2. However, in order to establish the preferential partner choice of TFs for the regulation of disease progression in both animals, we mainly consider the differences between the networks that were constructed for the chicken and duck tissues. Figure 4 shows the TFs and their partners in the regulatory events of these tissues, which are either found only in chicken or only in duck. In the following, we refer to a chicken∖duck network as the network of chicken TF cooperations without the duck TF cooperations and vice versa.

The greatest difference between chicken and duck can be observed in the chicken∖duck network for ileum 3 dpi, which contains 25 nodes and 14 edges (Figure 4B). Among the single nodes in this network, we found the ETS-related TF NERF and the bHLH heterodimeric TF AHR:ARNT. Interestingly, the lack of a partner indicates that the respective partner is present in the duck network, interacting with another TF. Such preferential partner choices are an indication of species-specific dimerization events, which form the basis for the regulation of different processes, such as immunity and inflammation [73]. Further ETS-related factors are found in the lung 1 and 3 dpi in the chicken∖duck networks (Figure 4C,D), and the monomer AHR is additionally found in the chicken∖duck network for ileum 1 dpi (Figure 4A). The importance of ETS-related and bHLH factors for chicken promoters was shown in the TF-enrichment (Section 3.1). Prominently, among all duck∖chicken networks, we observed different FOX and DLX homeo domain factors, which are not present in any chicken network. Both TF families were found to be enriched in the duck, but not the chicken promoters (see Section 3.1).

Remarkably, the differences in the cooperation networks are rather moderate in contrast to the divergent results of TF-enrichment between chicken and duck (Figure 3 and Figure 4). This indicates that, while single, enriched TFs in the promoter regions are rather species-specific, the TF-TF cooperation networks of both species share many common features and TF clusters seem to be preserved or classified by specific partner alterations.

### 3.3. Master Regulators

Functionally related genes involved in the same physiological or molecular processes, such as virus defense, are often coordinately regulated by the precise organization of TF binding [75]. This precise organization of TFs and their cooperation includes various upstream pathways forming complex regulatory network structures, in which different pathways can be connected in series, in parallel, or reverse, thus forming different feedforward or feedback loops [40]. One way to identify important regulators within such a complex regulatory network is the so-called “upstream analysis” [51], which aims to identify master regulators that are positioned at the top of the regulatory hierarchy and can be seen as common upstream regulators of a gene set, regulating the genes’ expression rates.

**Figure 5:**
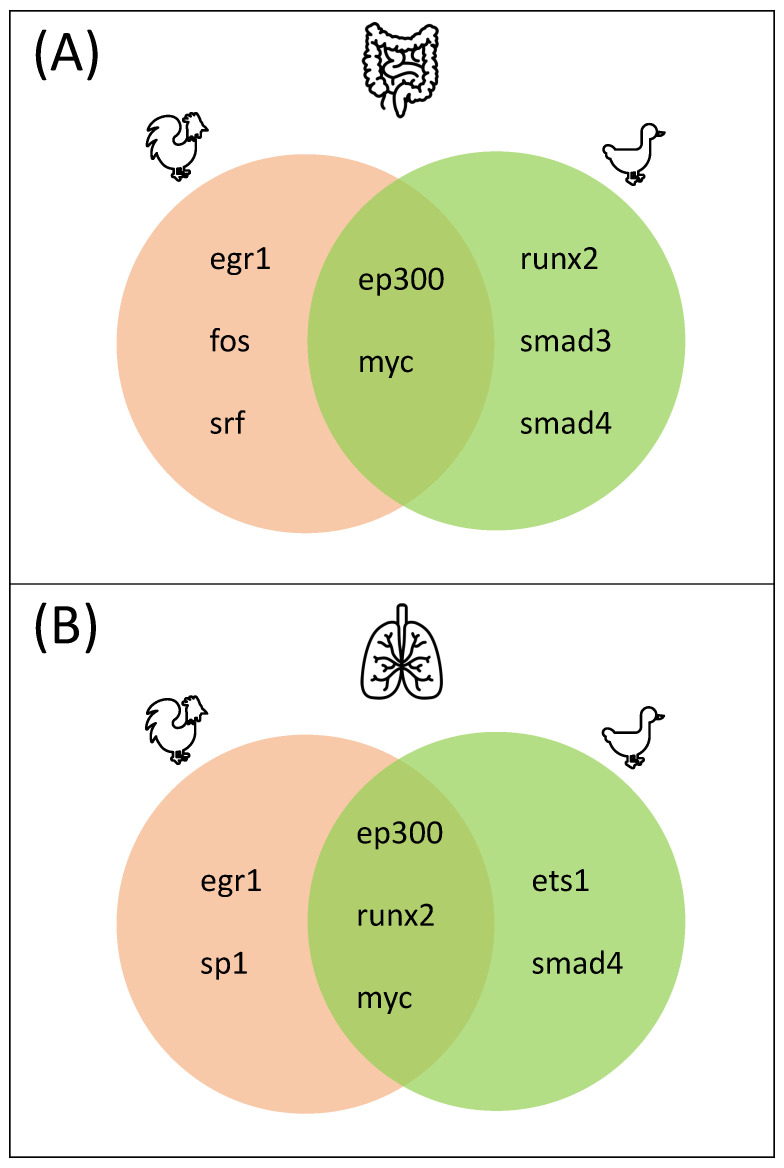
Common and species-specific master regulators for the (**A**) ileum and (**B**) lung tissue regulating the TF-TF cooperations.

During disease progression, the specific partner choices of TFs are of the utmost importance for an effective and rapid immune response [31,32,40]. Therefore, we mainly focus on the master regulators orchestrating the TF-TF cooperations in the following. The complete upstream regulatory networks based on the TF-TF cooperations can be obtained from Figure S4. Further, Figure 5 shows common and species-specific master regulators directing gene regulation in the lung and ileum tissues after infection with H5N1 (for both timepoints).

A closer look at the identified master regulators reveals that EGR1, SRF, FOS, and SP1 are unique to chicken in both tissues. EGR1 is considered a master transcription factor, regulating the expression of a range of genes involved in multiple cardiovascular diseases, such as atherosclerosis or ischemia in humans [76,77,78]. Furthermore, it is known to play various regulatory roles in processes such as cell death and survival or inflammatory processes [79]. In response to avian influenza in human, epithelial lung cells *EGR1*, as well as the chicken-specific master regulator gene *FOS*, were strongly downregulated [80].

The serum response factor (SRF) and the proto-oncogene factor FOS both play an important role in the inflammatory response after influenza infection in mammals [81]. The transcriptional regulator SRF first activates *FOS* expression [82,83], which encodes, together with *JUN*, the components of the transcription factor complex AP-1 [84,85]. AP-1 regulates a variety of processes such as cell proliferation and differentiation [84,85] but also activates the transcription of pro-inflammatory genes after an influenza infection [81]. In chicken trachea, *FOS* was shown to be upregulated after hydrogen-sulfide-induced oxidative stress, revealing the importance of FOS/IL8 signaling during tracheal inflammation [86]. Kim et al. [87] showed that a knockout of IRF7 in chicken DF-1 cells, and subsequent AIV infection, resulted in the altered gene expression pattern of several genes, including key immune response genes such as *IL12*, *FOS*, and *AP1*. The authors further suggest that this shift in expression pattern could be a compensation for the absence of IRF7 [87].

SP1 is involved in influenza A virus-induced mucin (i.e., MUC5AC) expression in mouse epithelial cells. Mucins, the gel-forming glycoproteins of mucus, are important to moisturize and protect surfaces from pathogens, and a mis- or overexpression of mucin may be related to various diseases, including different lung diseases caused by inflammation [88]. Furthermore, SP1 cooperates with different SMAD TFs in response to TGF-β, leading to the growth arrest of epithelial cells [89]. Interestingly, three SP family members (SP1-3) were found to be enriched in the chicken but not the duck promoters of the genes under study (Section 3.1).

In addition, the master regulators MYC and EP300 were identified as common to chicken and duck in both tissues. As an oncogenic TF, MYC is involved in several cellular processes related to cell growth, cell proliferation, or apoptosis [90]. Moreover, it is an important player in the JAK/STAT pathway, an important pathway in type I IFN response, as it is directly regulated by STAT TFs [9,71]. EP300 encodes a histone acetyltransferase that regulates the transcription of genes involved in cell proliferation and differentiation processes via chromatin remodeling. It is known to interact with a significant number of TFs, such as STAT, ETS1, and Ep53 in humans [91,92]. The importance of STAT in duck promoters has been shown in Section 3.1 and ETS1 acts as a master regulator in the lung tissue in duck. Furthermore, Leymarie et al. [93] observed that H5N1-infected mice developed a clear signature, leading to lung edema, which represents a pathogenic fluid accumulation in the lungs leading to respiratory dysfunction. Interestingly, they discovered an edema signature regulatory network consisting of different master TFs including EP300 and Runx1, a runt-related transcription factor [93]. Another Runx family member, Runx2, was identified as a common master regulator in the lung and as a duck-specific master regulator in the ileum. This finding enhances the importance of pathological edema-related processes during virus defense.

Master regulators that are unique to duck are of particular interest in our analysis, since they seem to activate pathways, leading to an effective differential expression of important genes, which is not the case for chicken. We identified three different duck-specific master regulators: ETS1 in the lung, SMAD3 in the ileum, and SMAD4 in both tissues.

As ETS factors play a role in both the innate and the adaptive immune response [64], they could be important master regulators controlling gene expression in duck HPAI defense. Among the ETS TFs, ETS1 and PU.1 seem to play the most important role in immunity in humans due to their control of immune cell development [64]. Surprisingly, different binding sites for ETS family members (except ETS1) have been identified as enriched in chicken but not duck promoters (see Section 3.1).

The importance of SMAD TFs in immune response and their tight link to the TGF-β and RLR-signaling pathways were revealed in Section 3.1. In particular, the SMAD3 family member is activated by TGF-β receptors and forms a transcriptional complex with SMAD4. The SMAD3/4 complex can then physically and functionally interact with c-Jun–c-Fos by binding to AP-1 binding sites to activate TGF-β responsible genes [62,94,95]. Hence, working in cooperation, the SMAD family members SMAD3 and 4, play a major role in TGF-β-mediated immune response and can be considered as promising targets for future studies.

In the second part of this section, we were additionally interested in the investigation of the master regulators targeting the enriched TFs of the DEG sets. As expected, the vast majority of the identified master regulators are unique to either chicken or duck in both tissues. The reason for this can be explained based on the distinct sets of enriched TFs presented in Section 3.1. Notably, the master regulators ARNT2 and EPAS1 were found only for chicken, while CRSP2, IRF9, and IRF7 were found only for duck. The complete upstream regulatory networks are provided in Figure S5. However, this finding does not reflect the assumption that the regulatory mechanisms of two orthologous gene sets share common features. Therefore, we presume that TF enrichment does not sufficiently represent the regulatory interplay underlying disease progression.

## 4. Conclusions

Until now, the mechanisms underlying the susceptibility of chicken and the effective immune response of duck are not completely understood. In this study, we performed a systematic analysis to investigate the transcriptional gene regulation underlying disease progression in ducks and chicken after infection with avian influenza. For this purpose, we identified upstream regulators, including TFs, their complex interplay, and master regulators, which are responsible for different immune responses in both species.

Our results suggest that there are major differences between the promoter regions of orthologous genes regarding the enrichment of TFs in both species. In particular, we identified promising TF families, which are important regulators of chicken (TF families such as SMAD, IRF, and ETS) or duck (TF families such as FOX, STAT, and POU). Although TF enrichment provides important insights, we could unravel the specific partner choice of TFs, which could be responsible for directing the different immune responses during disease progression. Subsequently, we applied a systems biology approach to identify common and species-specific master regulators. We found promising master regulators of duck genes in lung and ileum (RUNX2, SMAD3, SMAD4, and ETS1), which could be responsible for the duck’s effective differential gene expression in response to HPAI infection. Master regulators that were identified for the chicken orthologous gene set represent regulators that could be important for the effective regulation of gene expression after AIV infection, yet remain unsuccessful in living organisms. These master regulators include EGR1, FOS, SRF, and SP1, and could be interesting targets for future studies, since they could switch on several pathways targeting the genes that are important to the successful alleviation of HPAI infection. Based on our results, we highlight the importance of the RLR signaling, TGF-β, and the JAK/STAT pathways for virus defense in chickens and ducks. We are aware that the amount of mRNA does not necessarily reflect the amount of proteins that are available in living cells. For that reason, we emphasize the need for experimental data to assess protein availability, as well as the roles of master regulators and pathways in living organisms. To the best of our knowledge, there are no studies on altered immunity in duck after knockouts, overexpression or mutations in the identified upstream pathways. Therefore, knock-out, knock-in, or overexpression experiments in both chicken and duck would be of great interest. While this is beyond our current capabilities, it would be an important objective for future studies to investigate.

## Figures and Tables

**Table 1:** Numbers of differentially expressed genes (DEGs) in duck and chicken for the treatments with H5N1 (HPAI) and H5N2 (LPAI) virus after 1 and 3 days post-infection (dpi). The table is split into upregulated (LFC>0.58) and downregulated genes (LFC<−0.58).

Virus	Time	Tissue	Duck DEGs		Chicken DEGs	
			Upregulated	Downregulated	Upregulated	Downregulated
H5N1	1 dpi	lung	804	350	1	7
		ileum	193	63	5	6
	3 dpi	lung	605	486	1	0
		ileum	332	346	3	1
H5N2	1 dpi	lung	47	0	0	0
		ileum	42	1	20	2
	3 dpi	lung	1	0	0	0
		ileum	25	0	286	20

## Data Availability

Data and code for reproducing the analyses can be found at https://github.com/SelinaKlees/AIV_chicken_duck (accessed on 21 December 2021).

## Supplementary Materials

The following supporting information can be downloaded at: https://www.mdpi.com/article/10.3390/biology11020219/s1, Figure S1: Treemaps of the DEG sets regarding GO:Biological Processes of chicken and duck, Figure S2: Treemaps of the DEG sets regarding GO:Molecular Functions of chicken and duck, Figure S3: Full-size image of Figure 4, Figure S4: Schemes of the upstream regulatory networks revealing the top five master regulators of chicken and duck based on the TF-TF cooperation results, Figure S5: Schemes of the upstream regulatory networks revealing the top five master regulators of chicken and duck based on the TF enrichment results, Table S1: DEG sets for all experimental conditions, Table S2: GO-term enrichment of all DEG sets of chicken and duck, Table S3: enriched TFBSs in different promoter sets of chicken and duck, Table S4: TF cooperation networks of chicken and duck, File S1: PWMs included in the custom avian PWM profile, File S2: Cytoskape session of TF cooperation networks as .cys file.

## Abbreviations

The following abbreviations are used in this manuscript:

AIV	Avian influenza virus
HPAI	High pathogenic avian influenza
LPAI	Low pathogenic avian influenza
dpi	Days post infection
DEG	Differentially expressed gene
TF	Transcription factor
TFBS	Transcription factor binding site
TSS	Transcription start site
BP	Base pairs
FDR	False discovery rate
LFC	Log${}_{2}$ fold change
GO	Gene Ontology
PMI	Pointwise mutual information
PWM	Position weight matrix
IFN	Interferon
IRF	Interferon regulatory factor
ISG	Interferon-stimulated gene
STAT	Signal transducer and activator of transcription
RLR	RIG-I-like receptor
FOX	Forkhead box
HOX	Homeobox
JAK	Janus–kinase
