# Supplementary material for: Comparative Investigation of Gene Regulatory Processes Underlying Avian Influenza Viruses in Chicken and Duck
Source: Biology (Basel). 2022 Jan 29;11(2):219. doi: 10.3390/biology11020219 (PMC8868632; doi:10.3390/biology11020219)
Supplement: Supplementary file 1 [file biology-11-00219-s001.zip › Supplementary_Figure_S1_treemaps_BP.pdf]

Supplementary Figures S1: Treemaps for functionally enriched (P < 0.05) Gene Ontology (GO) terms of the differentially expressed gene (DEG) sets separated into up- and downregualted DEGs of chickens and ducks. Treemaps of experimental conditions that are not shown here did not deliver any enriched GO-terms. Source = GO: Biological Processes

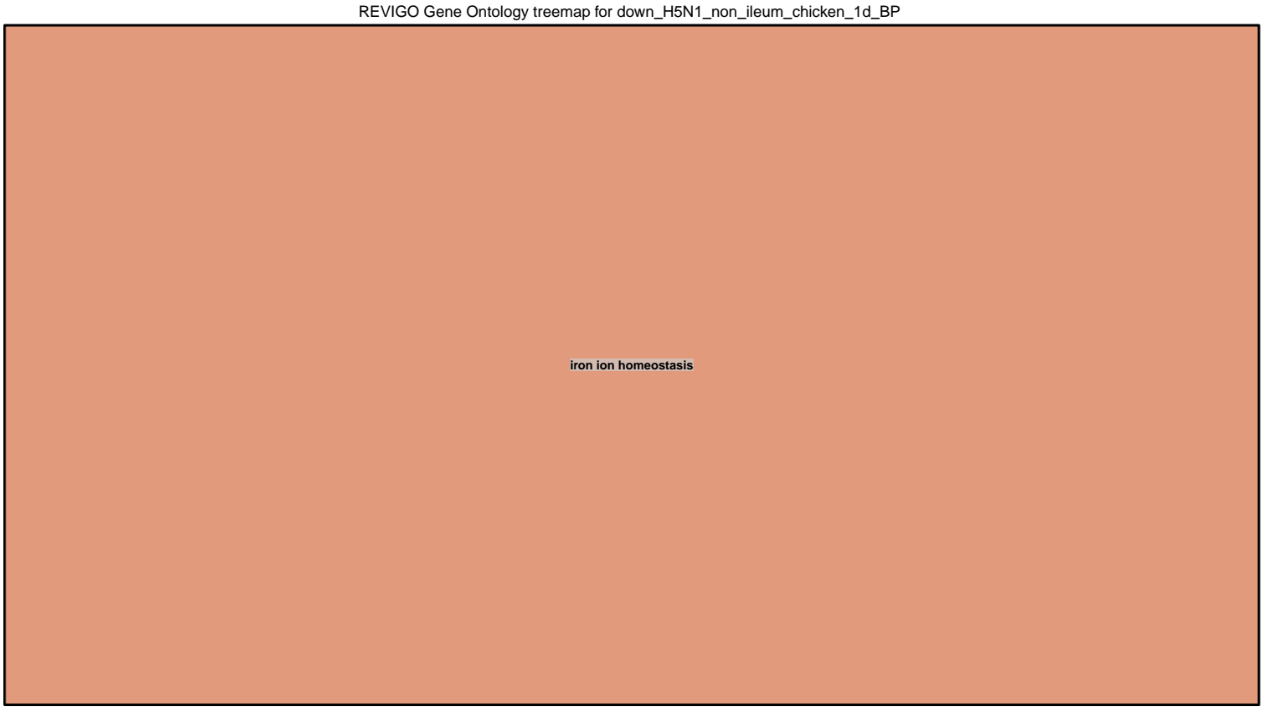

Figure S1.1: GO treemap for functionally enriched (P < 0.05) downregulated DEGs derived under the following experimental condition: chickens infected with H5N1 vs. mock-infected control measured in the ileum at 1 day post-infection (dpi). The boxes are grouped together based on the upper-hierarchy GO-term which is written in bold letters. Source = GO: Biological Processes

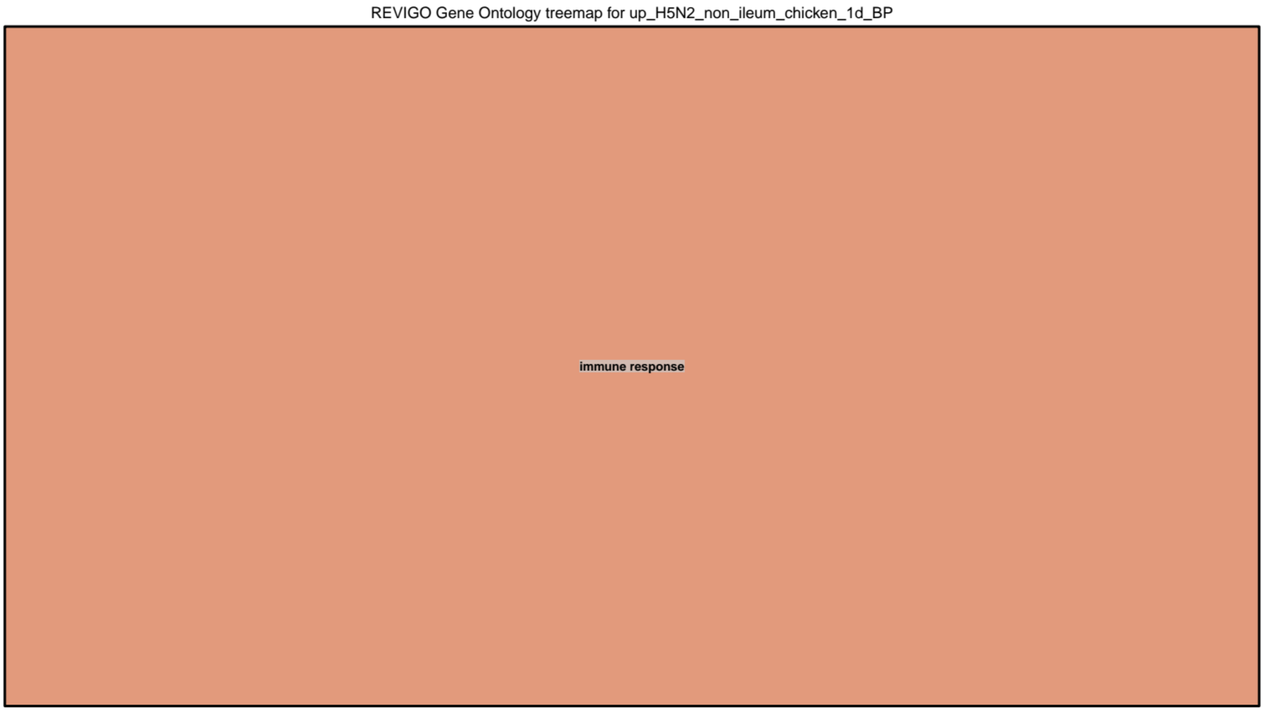

Figure S1.2: GO treemap for functionally enriched (P < 0.05) upregulated DEGs derived under the following experimental condition: chickens infected with H5N2 vs. mock-infected control measured in the ileum after 1 dpi. The boxes are grouped together based on the upper-hierarchy GO-term which is written in bold letters. Source = GO: Biological Processes

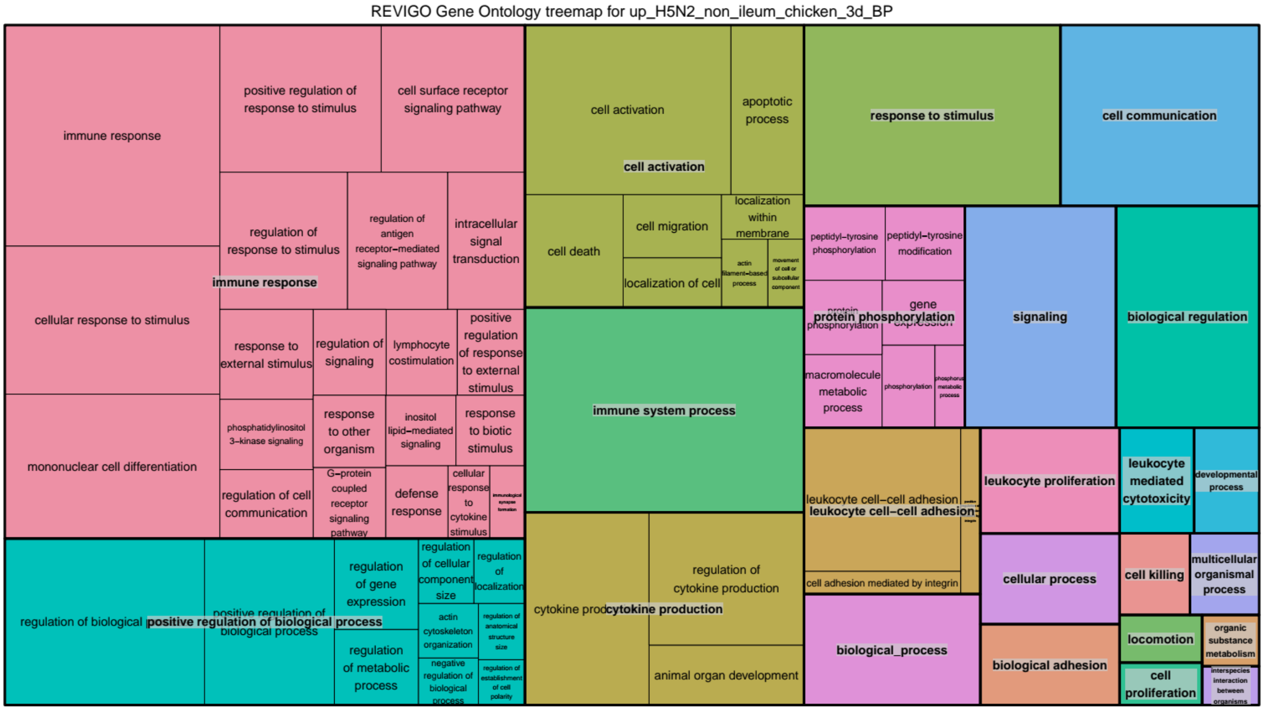

Figure S1.3: GO treemap for for functionally enriched (P < 0.05) upregulated DEGs derived under the following experimental condition: chickens infected with H5N2 vs. mock-infected control measured in the ileum after 3 dpi. The boxes are grouped together based on the upper-hierarchy GO-term which is written in bold letters. Source = GO: Biological Processes

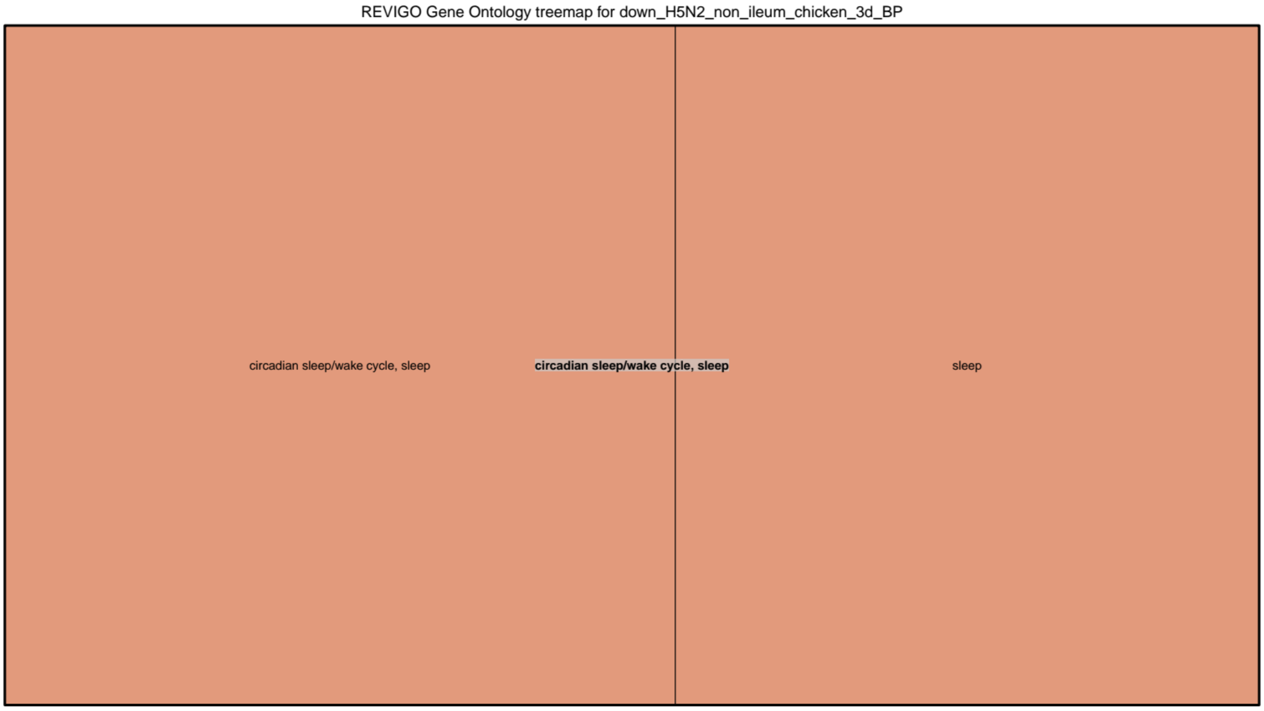

Figure S1.4: GO treemap for for functionally enriched (P < 0.05) downregulated DEGs derived under the following experimental condition: chickens infected with H5N2 vs. mock-infected control measured in the ileum after 3 dpi. The boxes are grouped together based on the upper-hierarchy GO-term which is written in bold letters. Source = GO: Biological Processes

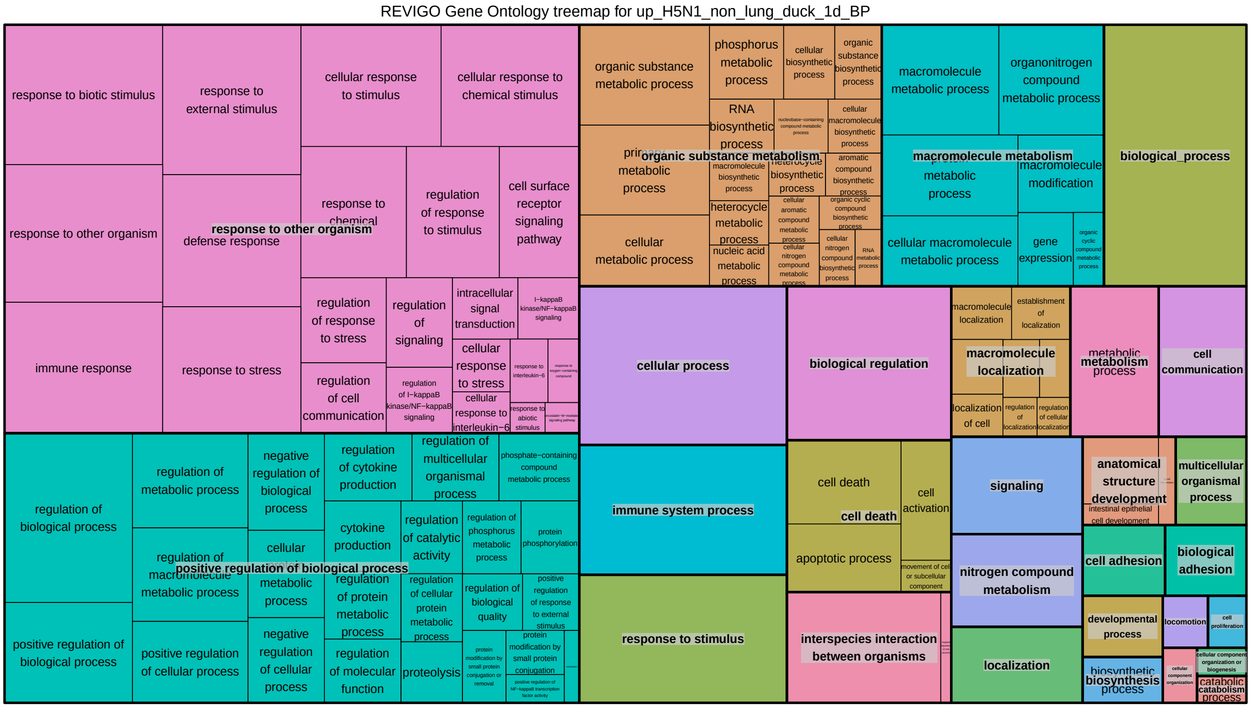

Figure S1.5: GO treemap for for functionally enriched (P < 0.05) upregulated DEGs derived under the following experimental condition: ducks infected with H5N1 vs. mock-infected control measured in the lung after 1 dpi. The boxes are grouped together based on the upper-hierarchy GO-term which is written in bold letters. Source = GO: Biological Processes

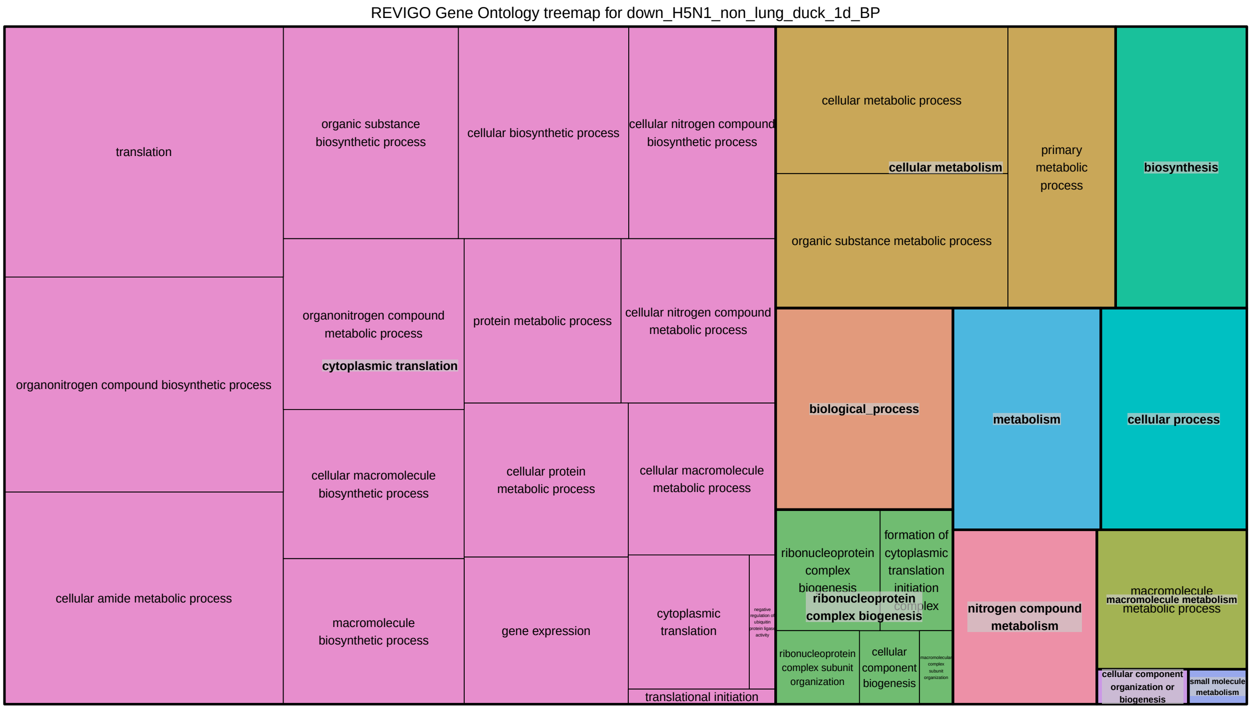

Figure S1.6: GO treemap for for functionally enriched (P < 0.05) downregulated DEGs derived under the following experimental condition: ducks infected with H5N1 vs. mock-infected control measured in the lung after 1 dpi. The boxes are grouped together based on the upper-hierarchy GO-term which is written in bold letters. Source = GO: Biological Processes

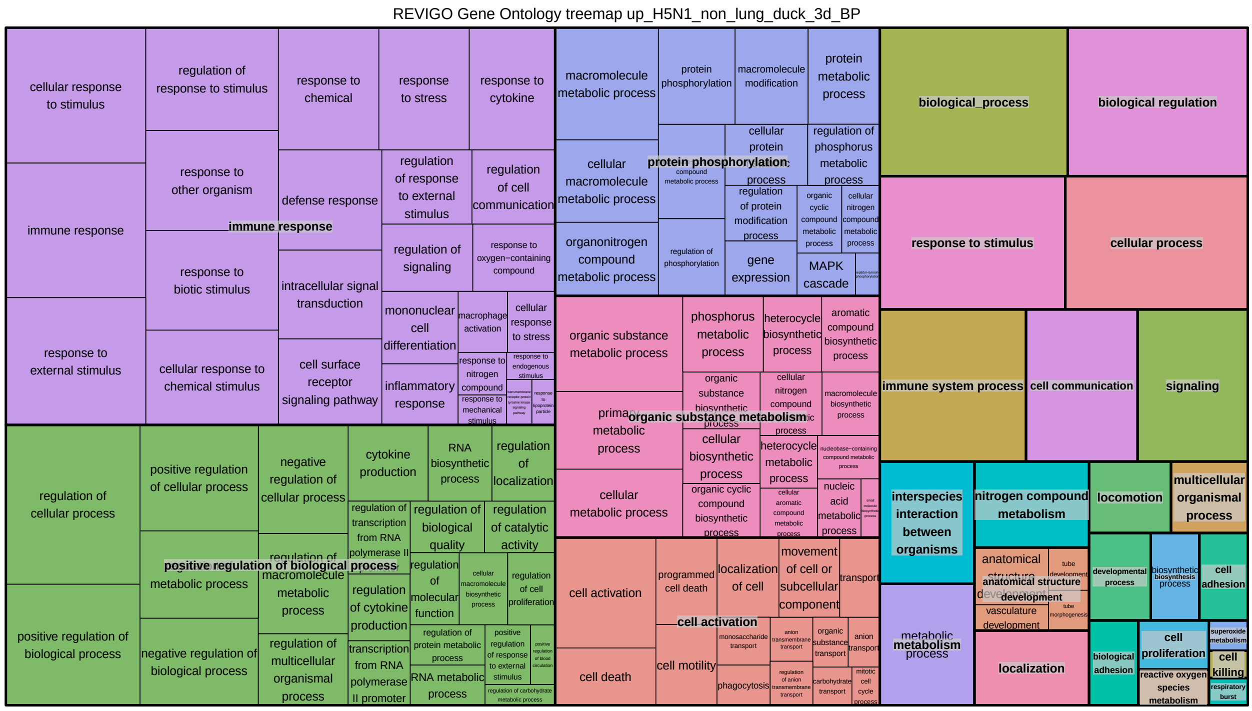

Figure S1.7: GO treemap for for functionally enriched (P < 0.05) upregulated DEGs derived under the following experimental condition: ducks infected with H5N1 vs. mock-infected control measured in the lung after 3 dpi. The boxes are grouped together based on the upper-hierarchy GO-term which is written in bold letters. Source = GO: Biological Processes

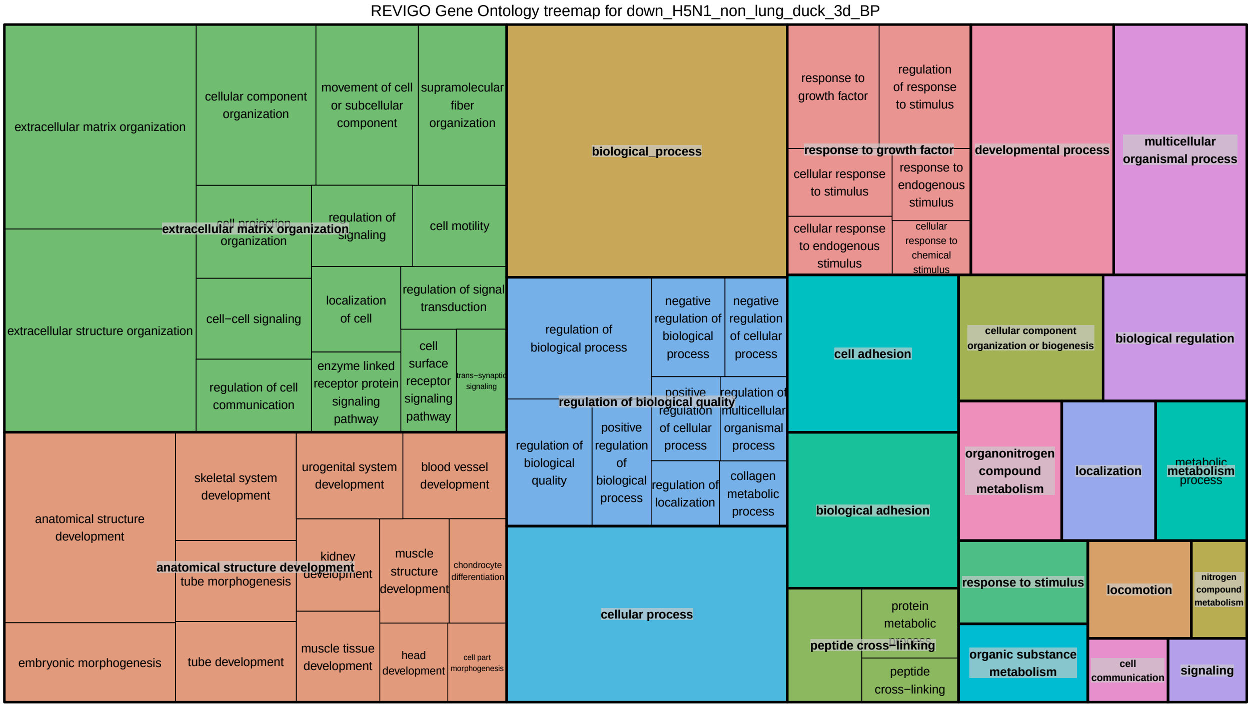

Figure S1.8: GO treemap for for functionally enriched (P < 0.05) downregulated DEGs derived under the following experimental condition: ducks infected with H5N1 vs. mock-infected control measured in the lung after 3 dpi. The boxes are grouped together based on the upper-hierarchy GO-term which is written in bold letters. Source = GO: Biological Processes

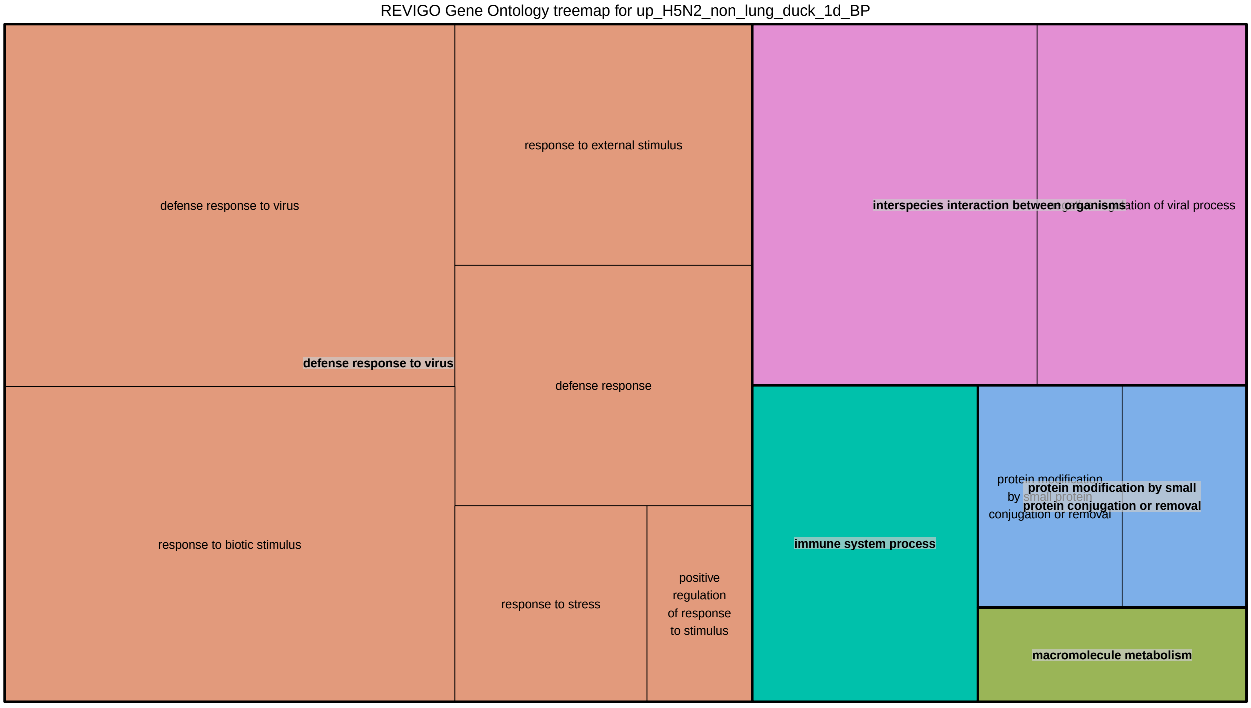

Figure S1.9: GO treemap for for functionally enriched (P < 0.05) upregulated DEGs derived under the following experimental condition: ducks infected with H5N2 vs. mock-infected control measured in the lung after 1 dpi. The boxes are grouped together based on the upper-hierarchy GO-term which is written in bold letters. Source = GO: Biological Processes

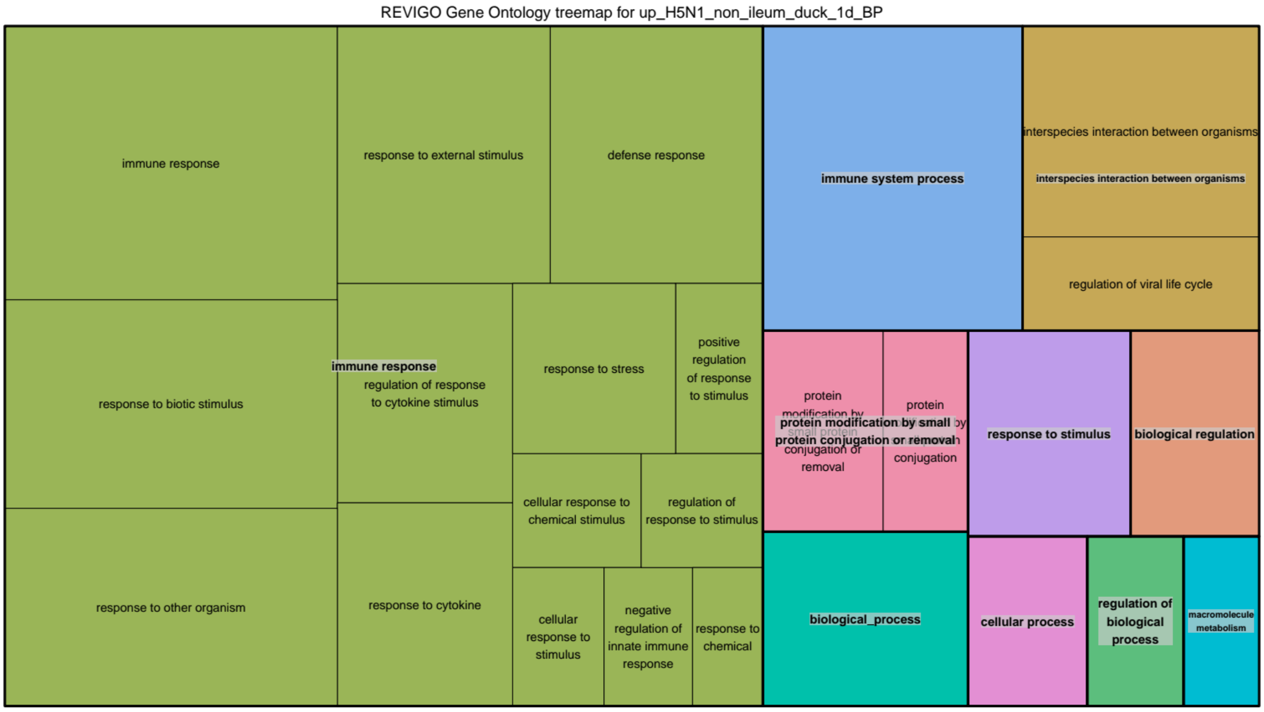

Figure S1.10: GO treemap for for functionally enriched (P < 0.05) upregulated DEGs derived under the following experimental condition: ducks infected with H5N1 vs. mock-infected control measured in the ileum after 1 dpi. The boxes are grouped together based on the upper-hierarchy GO-term which is written in bold letters. Source = GO: Biological Processes

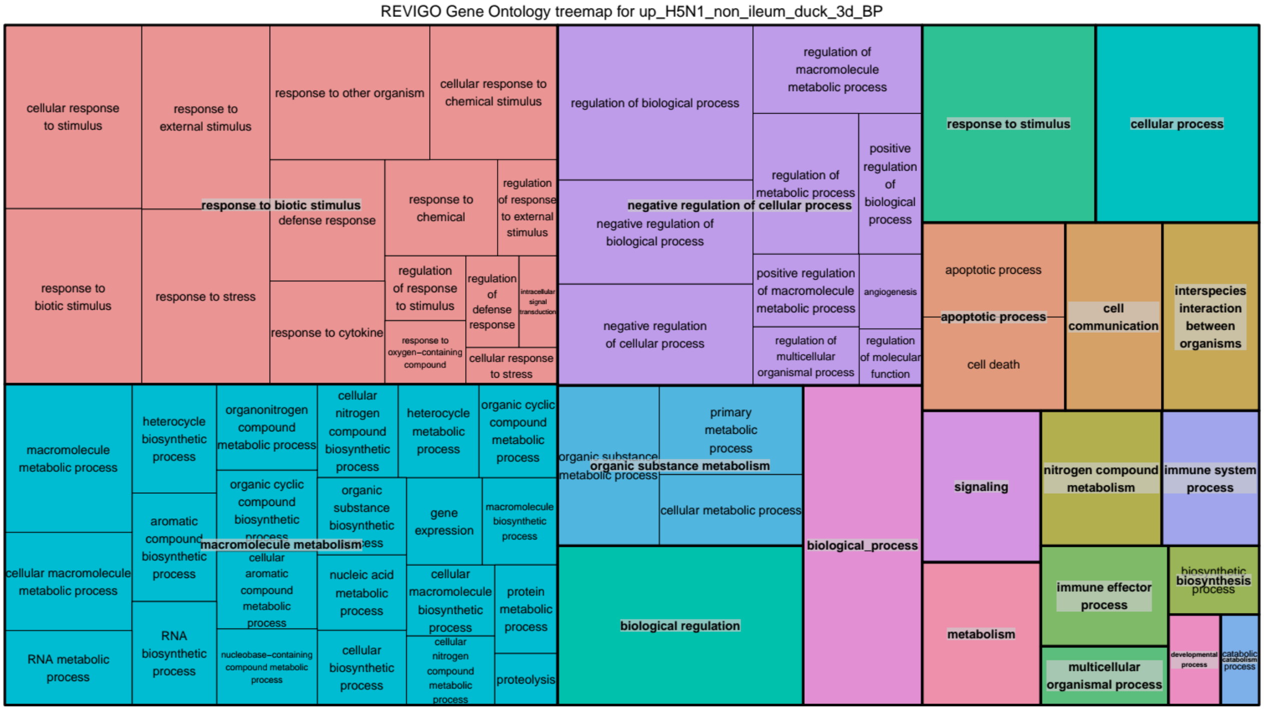

Figure S1.11: GO treemap for for functionally enriched (P < 0.05) upregulated DEGs derived under the following experimental condition: ducks infected with H5N1 vs. mock-infected control measured in the ileum after 3 dpi. The boxes are grouped together based on the upper-hierarchy GO-term which is written in bold letters. Source = GO: Biological Processes

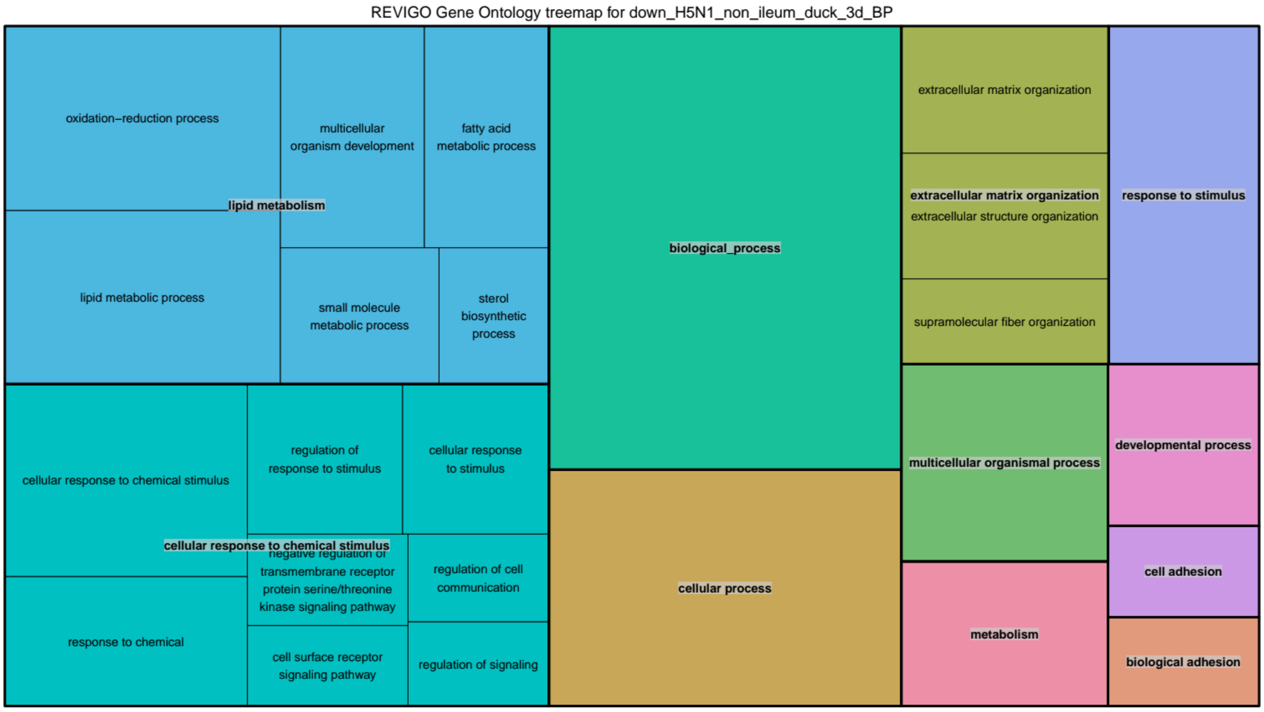

Figure S1.12: GO treemap for for functionally enriched (P < 0.05) downregulated DEGs derived under the following experimental condition: ducks infected with H5N1 vs. mock-infected control measured in the ileum after 3 dpi. The boxes are grouped together based on the upper-hierarchy GO-term which is written in bold letters. Source = GO: Biological Processes

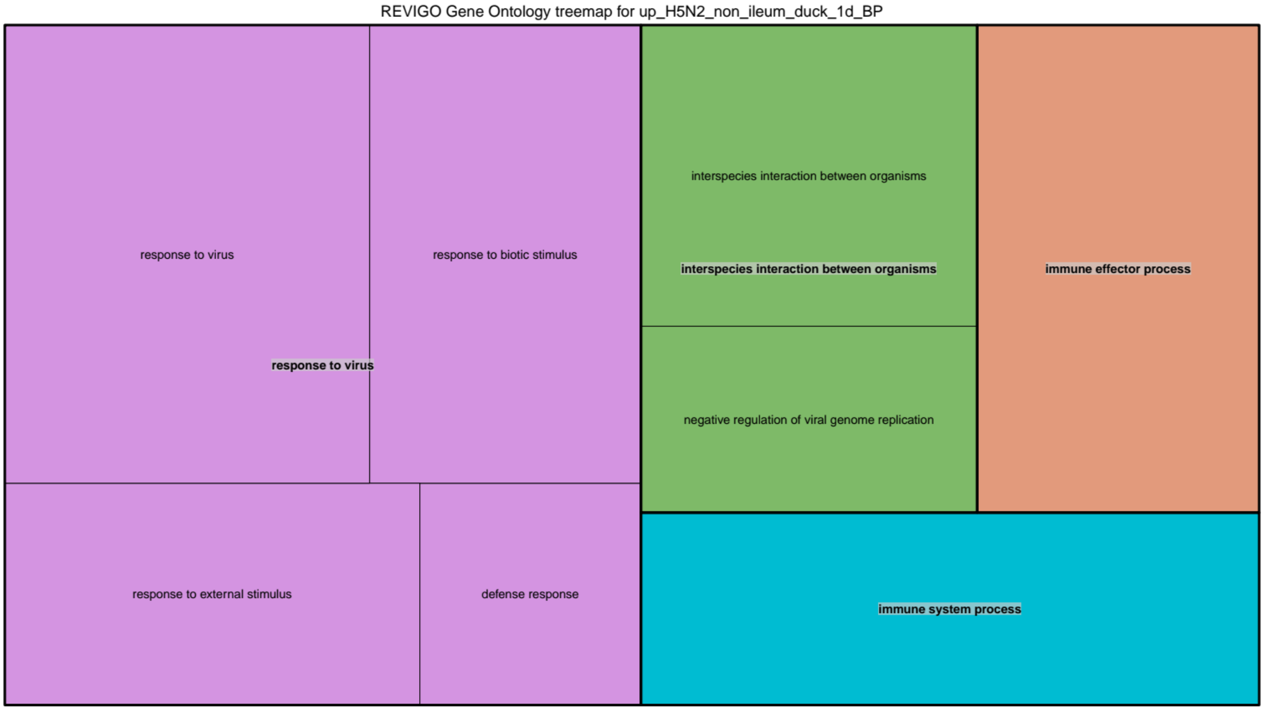

Figure S1.13: GO treemap for for functionally enriched (P < 0.05) upregulated DEGs derived under the following experimental condition: ducks infected with H5N2 vs. mock-infected control measured in the ileum after 1 dpi. The boxes are grouped together based on the upper-hierarchy GO-term which is written in bold letters. Source = GO: Biological Processes

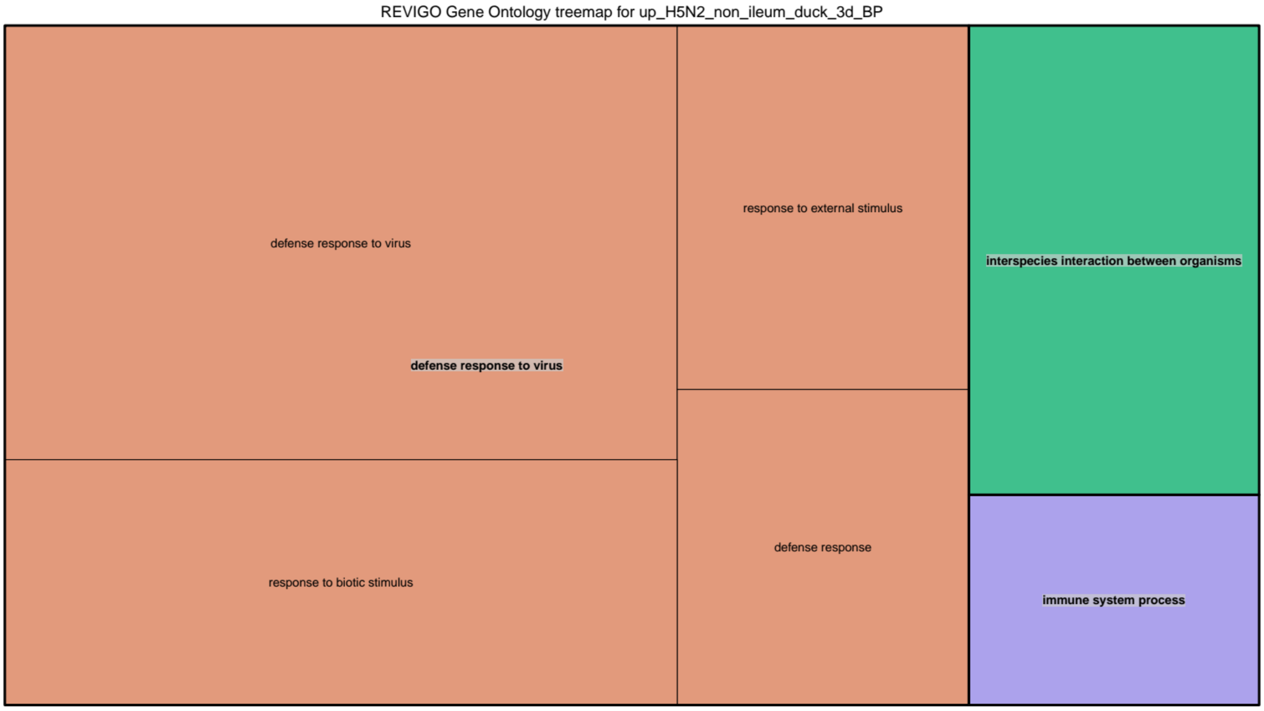

Figure S1.14: GO treemap for for functionally enriched (P < 0.05) upregulated DEGs derived under the following experimental condition: ducks infected with H5N2 vs. mock-infected control measured in the ileum after 3 dpi. The boxes are grouped together based on the upper-hierarchy GO-term which is written in bold letters. Source = GO: Biological Processes
