# Supplementary material for: Comparative Investigation of Gene Regulatory Processes Underlying Avian Influenza Viruses in Chicken and Duck
Source: Biology (Basel). 2022 Jan 29;11(2):219. doi: 10.3390/biology11020219 (PMC8868632; doi:10.3390/biology11020219)
Supplement: Supplementary file 1 [file biology-11-00219-s001.zip › Supplementary_Figure_S2_treemaps_MF.pdf]

Supplementary Figures S2: Treemaps for functionally enriched (P < 0.05) Gene Ontology (GO) terms of the differentially expressed gene (DEG) sets separated into up- and downregualted DEGs of chickens and ducks. Treemaps of experimental conditions that are not shown here did not deliver any enriched GO-terms. Source = GO: Molecular functions

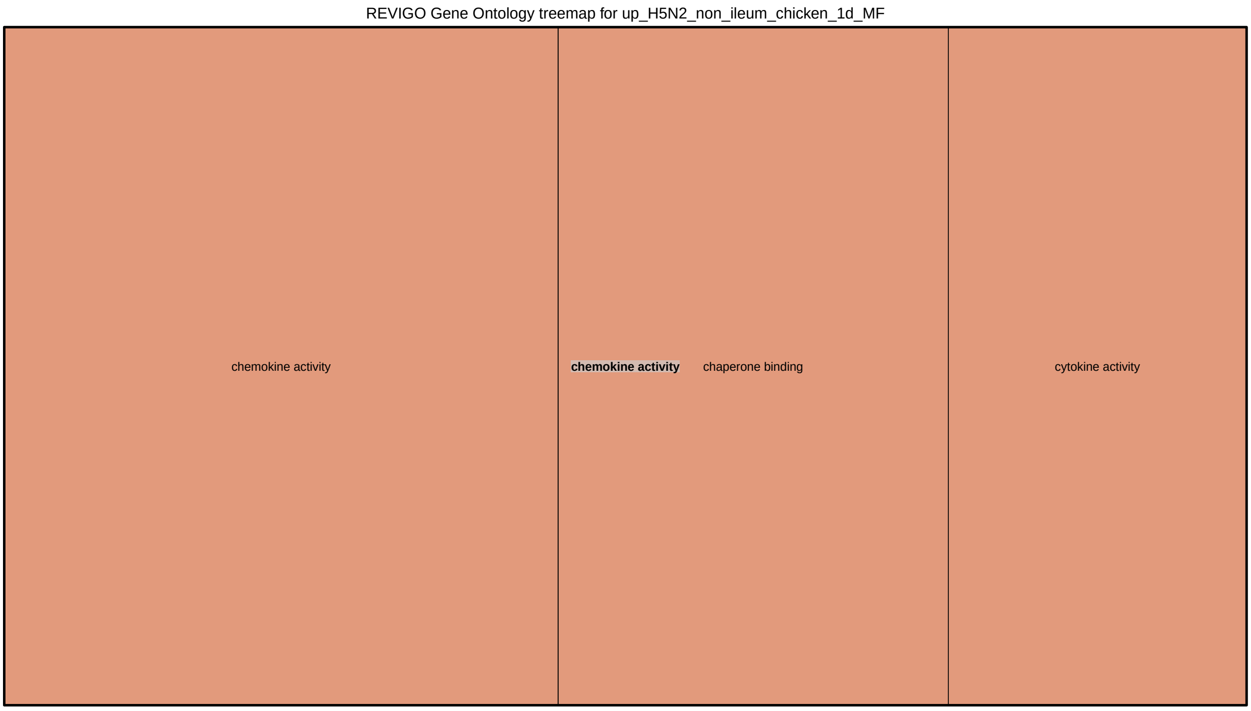

Figure S2.1: GO treemap for functionally enriched (P < 0.05) upregulated DEGs derived under the following experimental condition: chickens infected with H5N2 vs. mock-infected control measured in the ileum at 1 day post-infection (dpi). The boxes are grouped together based on the upper-hierarchy GO-term which is written in bold letters. Source = GO: Molecular functions

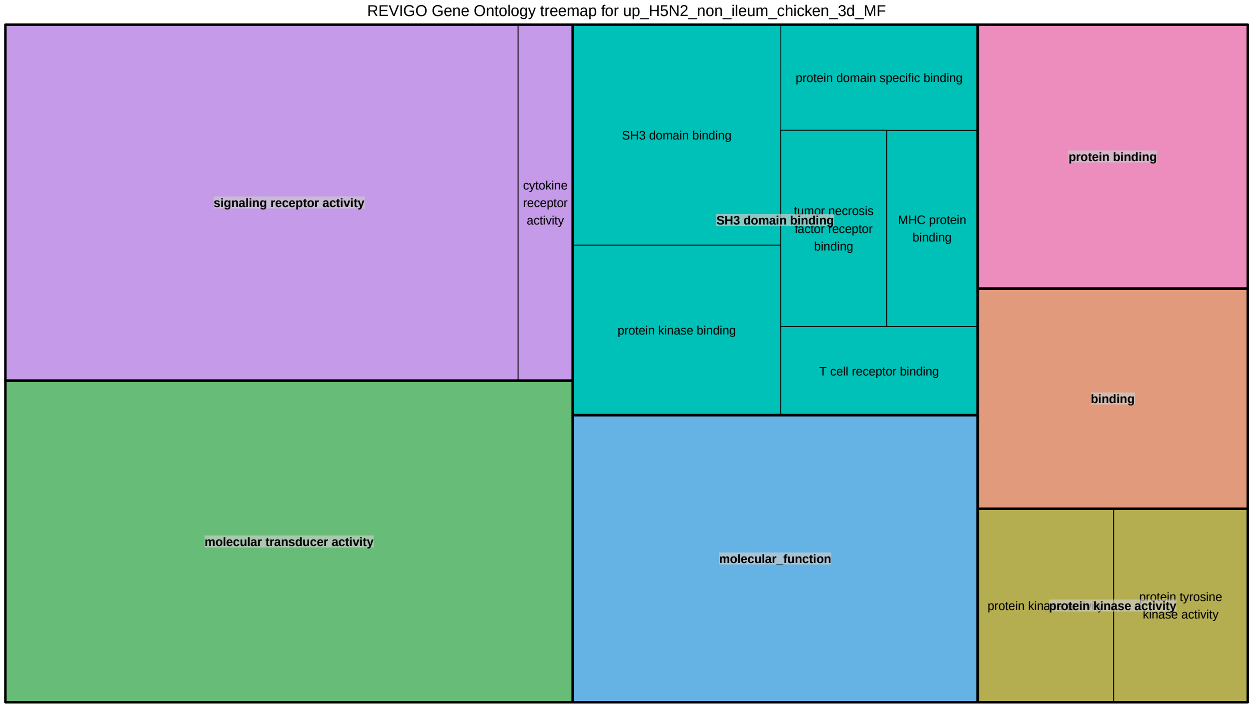

Figure S2.2: GO treemap for functionally enriched (P < 0.05) upregulated DEGs derived under the following experimental condition: chickens infected with H5N2 vs. mock-infected control measured in the ileum at 3 dpi. The boxes are grouped together based on the upper-hierarchy GO-term which is written in bold letters. Source = GO: Molecular functions

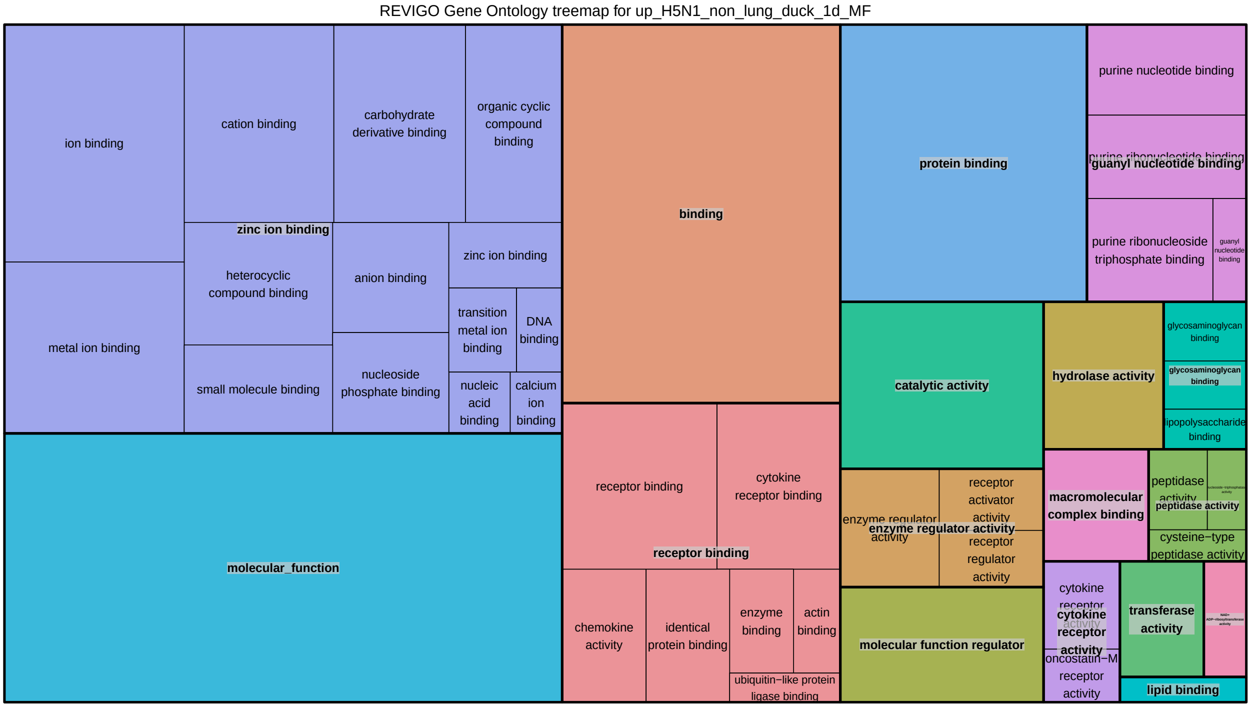

Figure S2.3: GO treemap for functionally enriched (P < 0.05) upregulated DEGs derived under the following experimental condition: ducks infected with H5N1 vs. mock-infected control measured in the lung at 1 dpi. The boxes are grouped together based on the upper-hierarchy GO-term which is written in bold letters. Source = GO: Molecular functions

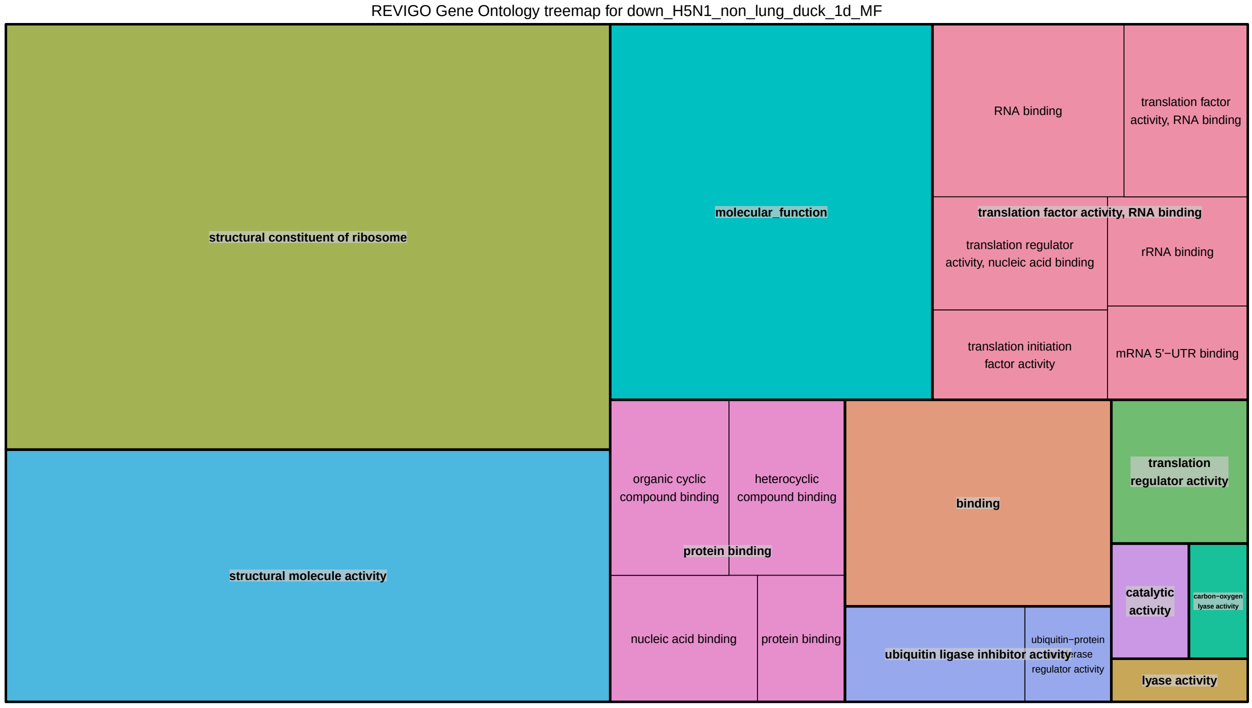

Figure S2.4: GO treemap for functionally enriched (P < 0.05) downregulated DEGs derived under the following experimental condition: ducks infected with H5N1 vs. mock-infected control measured in the lung at 1 dpi. The boxes are grouped together based on the upper-hierarchy GO-term which is written in bold letters. Source = GO: Molecular functions

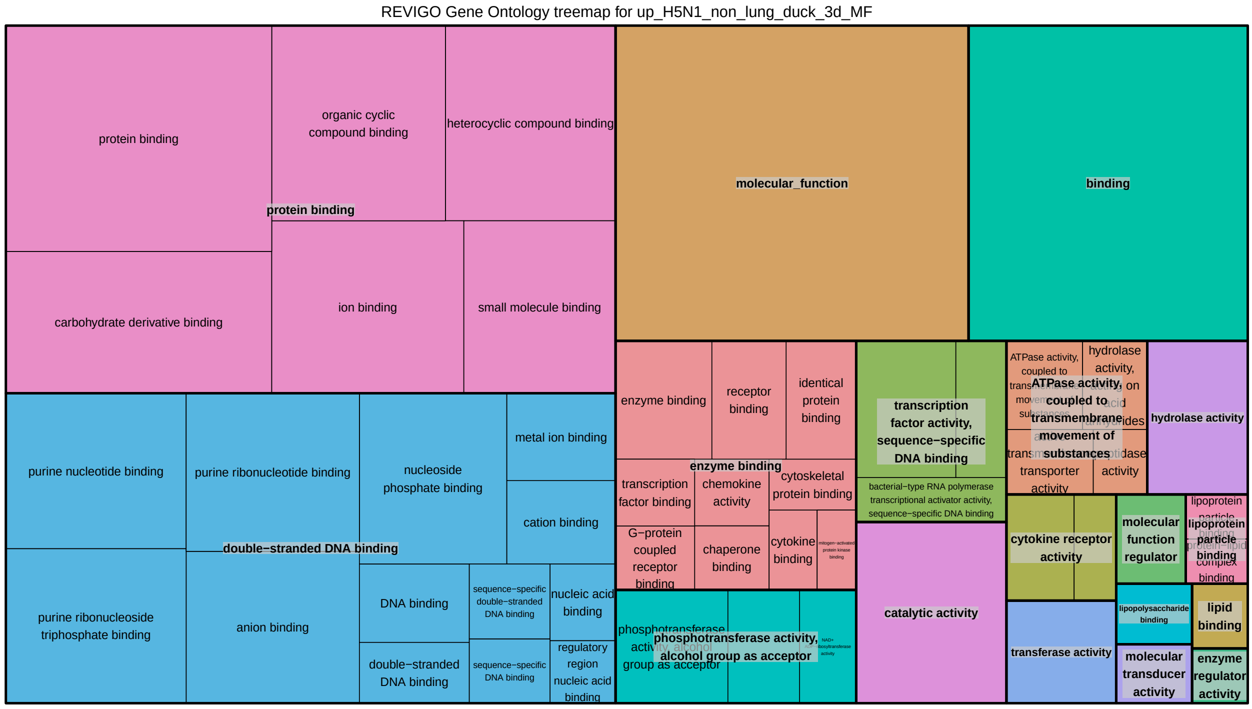

Figure S2.5: GO treemap for functionally enriched (P < 0.05) upregulated DEGs derived under the following experimental condition: ducks infected with H5N1 vs. mock-infected control measured in the lung at 3 dpi. The boxes are grouped together based on the upper-hierarchy GO-term which is written in bold letters. Source = GO: Molecular functions

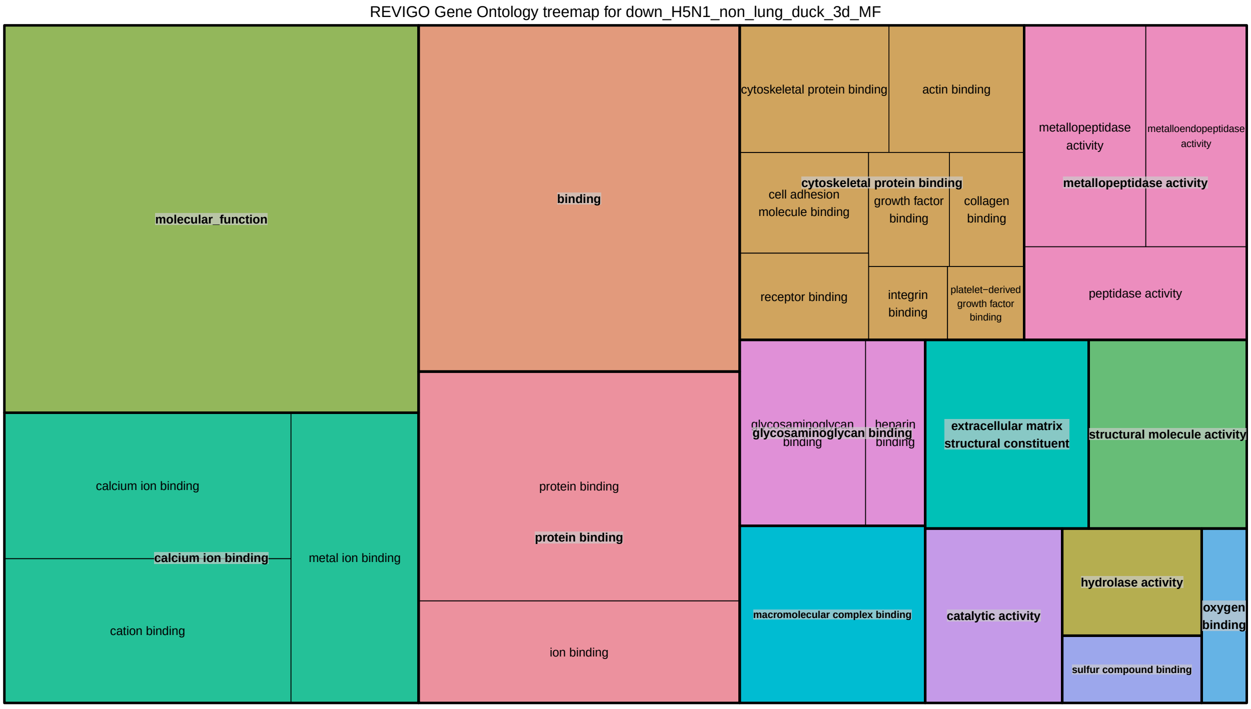

Figure S2.6: GO treemap for functionally enriched (P < 0.05) downregulated DEGs derived under the following experimental condition: ducks infected with H5N1 vs. mock-infected control measured in the lung at 3 dpi. The boxes are grouped together based on the upper-hierarchy GO-term which is written in bold letters. Source = GO: Molecular functions

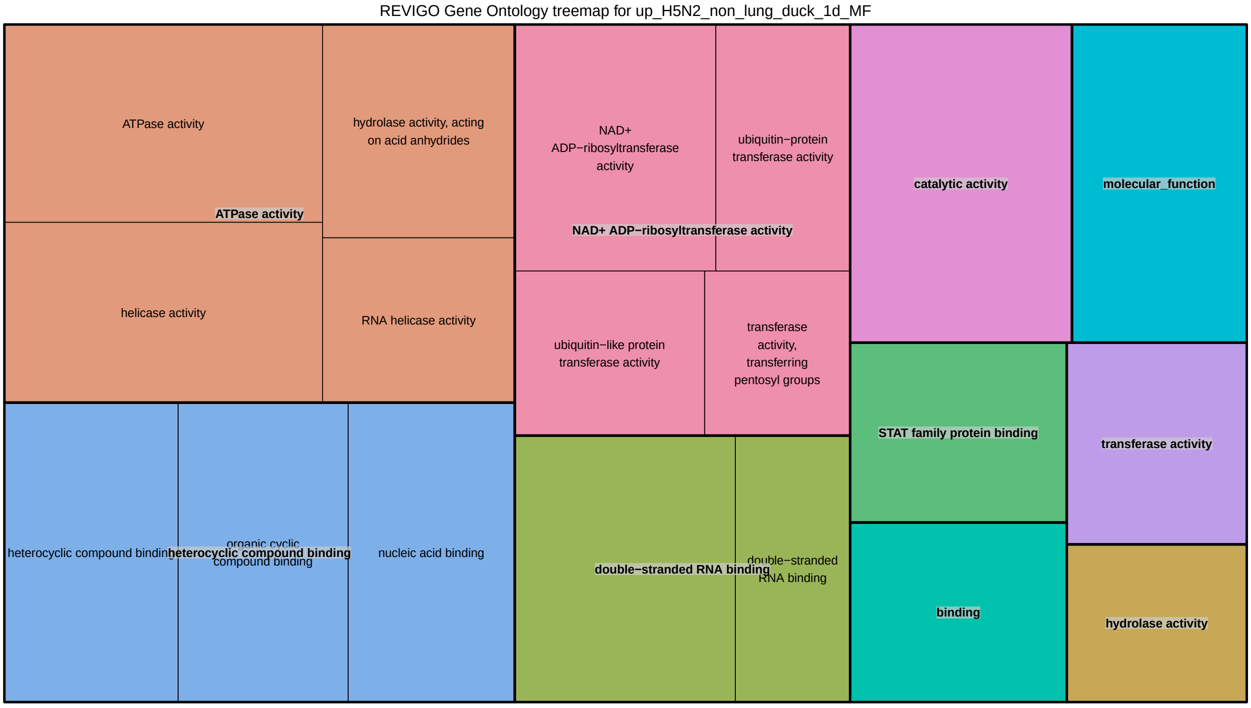

Figure S2.7: GO treemap for functionally enriched (P < 0.05) upregulated DEGs derived under the following experimental condition: ducks infected with H5N2 vs. mock-infected control measured in the lung at 1 dpi. The boxes are grouped together based on the upper-hierarchy GO-term which is written in bold letters. Source = GO: Molecular functions

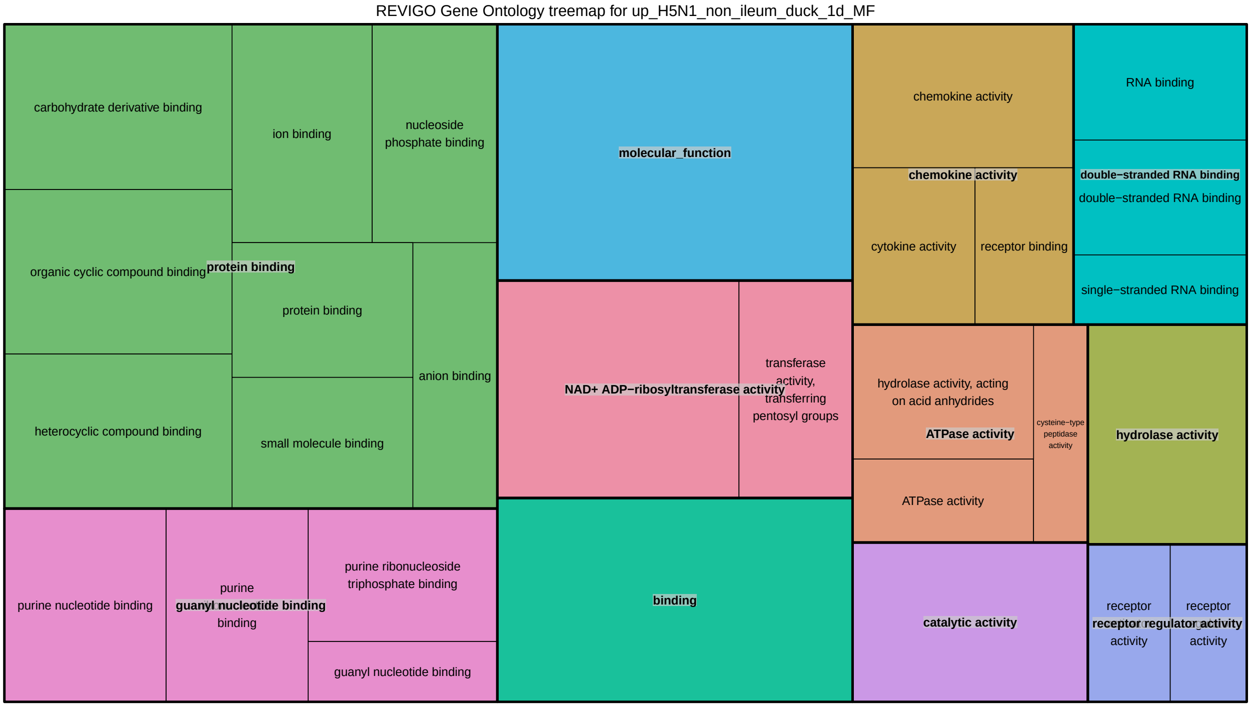

Figure S2.8: GO treemap for functionally enriched (P < 0.05) upregulated DEGs derived under the following experimental condition: ducks infected with H5N1 vs. mock-infected control measured in the ileum at 1 dpi. The boxes are grouped together based on the upper-hierarchy GO-term which is written in bold letters. Source = GO: Molecular functions

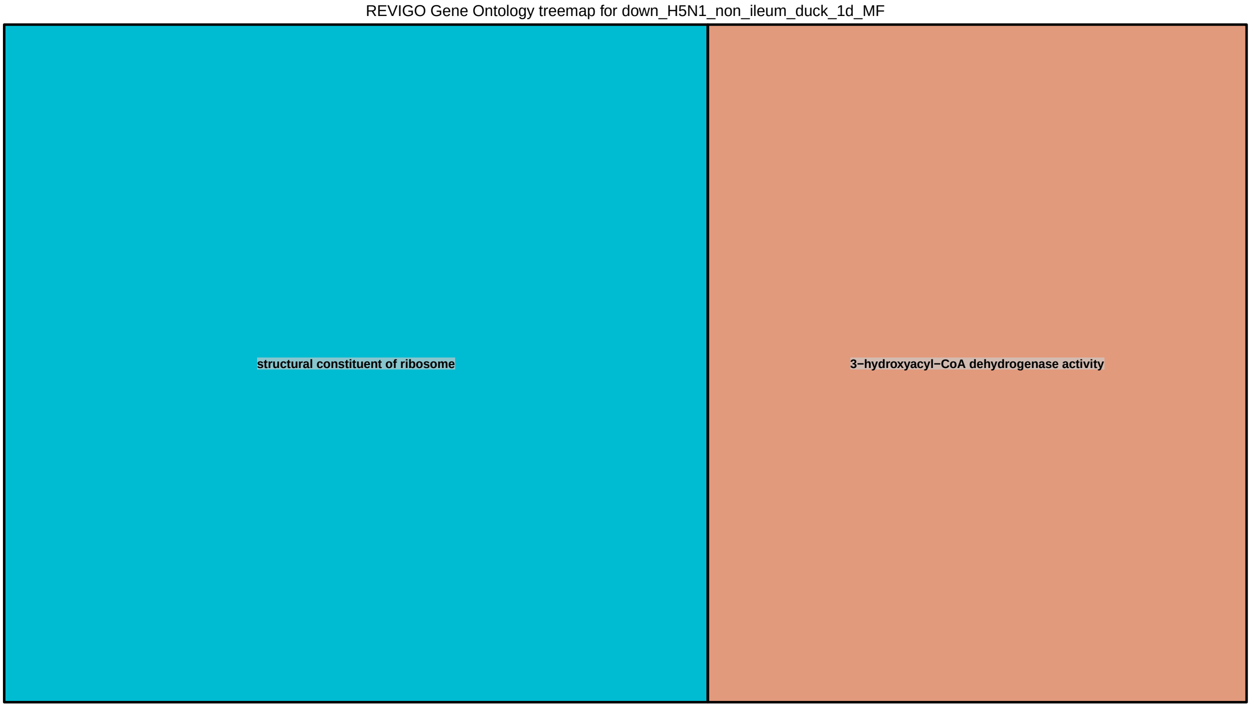

Figure S2.9: GO treemap for functionally enriched (P < 0.05) downregulated DEGs derived under the following experimental condition: ducks infected with H5N1 vs. mock-infected control measured in the ileum at 1 dpi. The boxes are grouped together based on the upper-hierarchy GO-term which is written in bold letters. Source = GO: Molecular functions

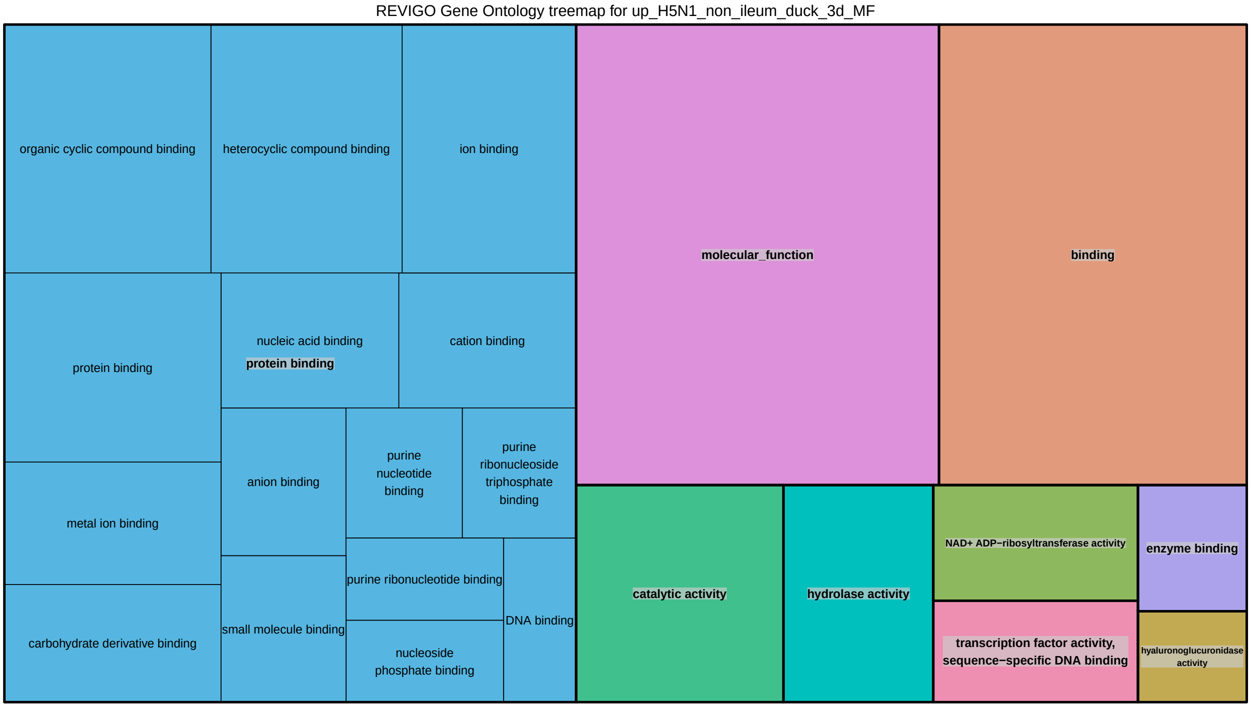

Figure S2.10: GO treemap for functionally enriched (P < 0.05) upregulated DEGs derived under the following experimental condition: ducks infected with H5N1 vs. mock-infected control measured in the ileum at 3 dpi. The boxes are grouped together based on the upper-hierarchy GO-term which is written in bold letters. Source = GO: Molecular functions

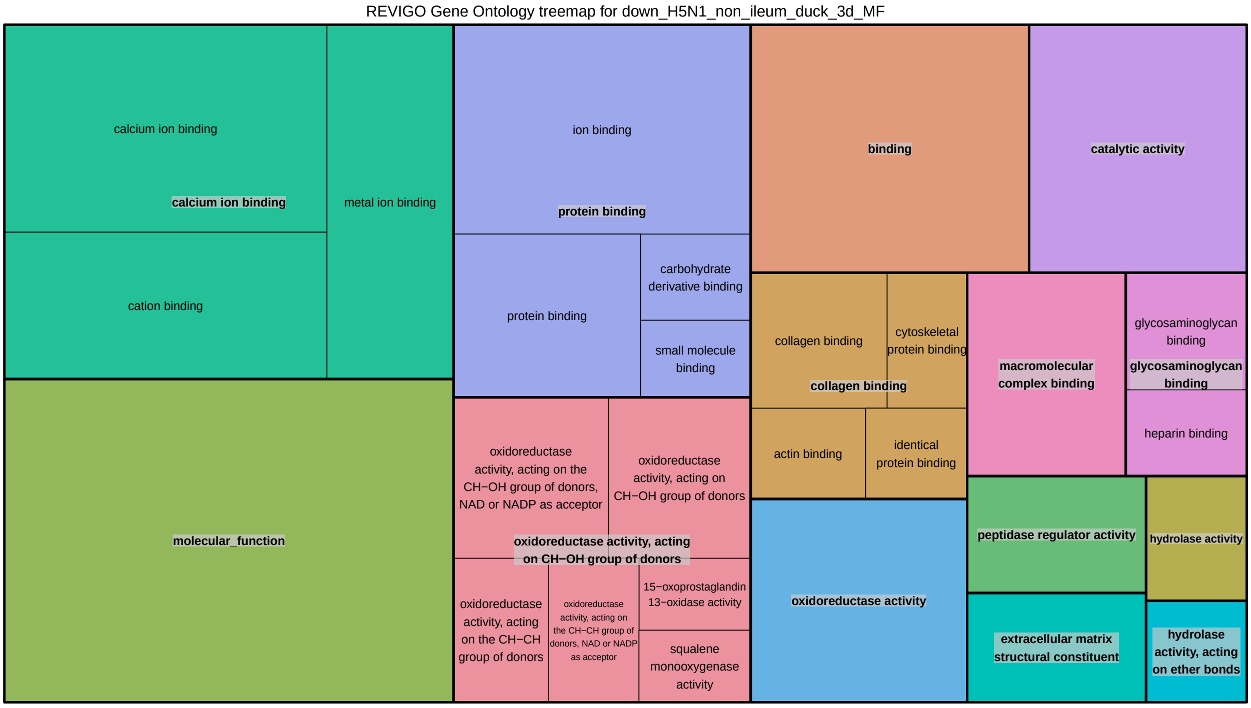

Figure S2.11: GO treemap for functionally enriched (P < 0.05) downregulated DEGs derived under the following experimental condition: ducks infected with H5N1 vs. mock-infected control measured in the ileum at 3 dpi. The boxes are grouped together based on the upper-hierarchy GO-term which is written in bold letters. Source = GO: Molecular functions

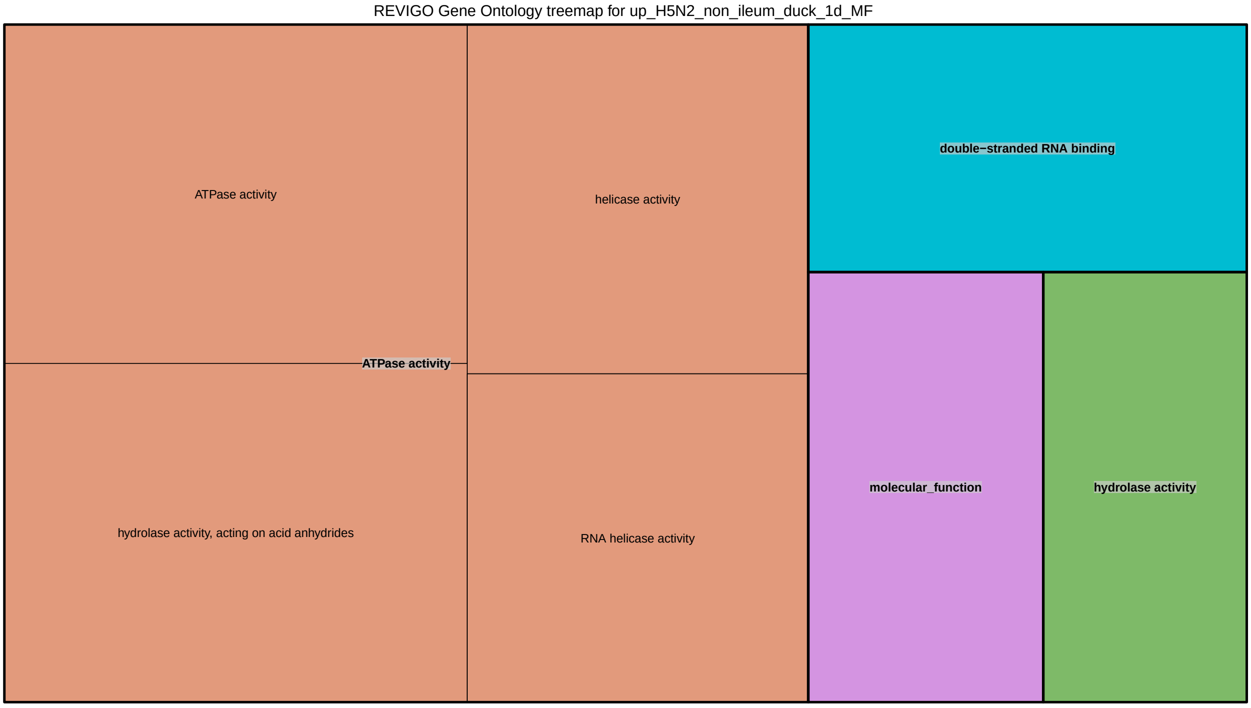

Figure S2.12: GO treemap for functionally enriched (P < 0.05) upregulated DEGs derived under the following experimental condition: ducks infected with H5N2 vs. mock-infected control measured in the ileum at 1 dpi. The boxes are grouped together based on the upper-hierarchy GO-term which is written in bold letters. Source = GO: Molecular functions

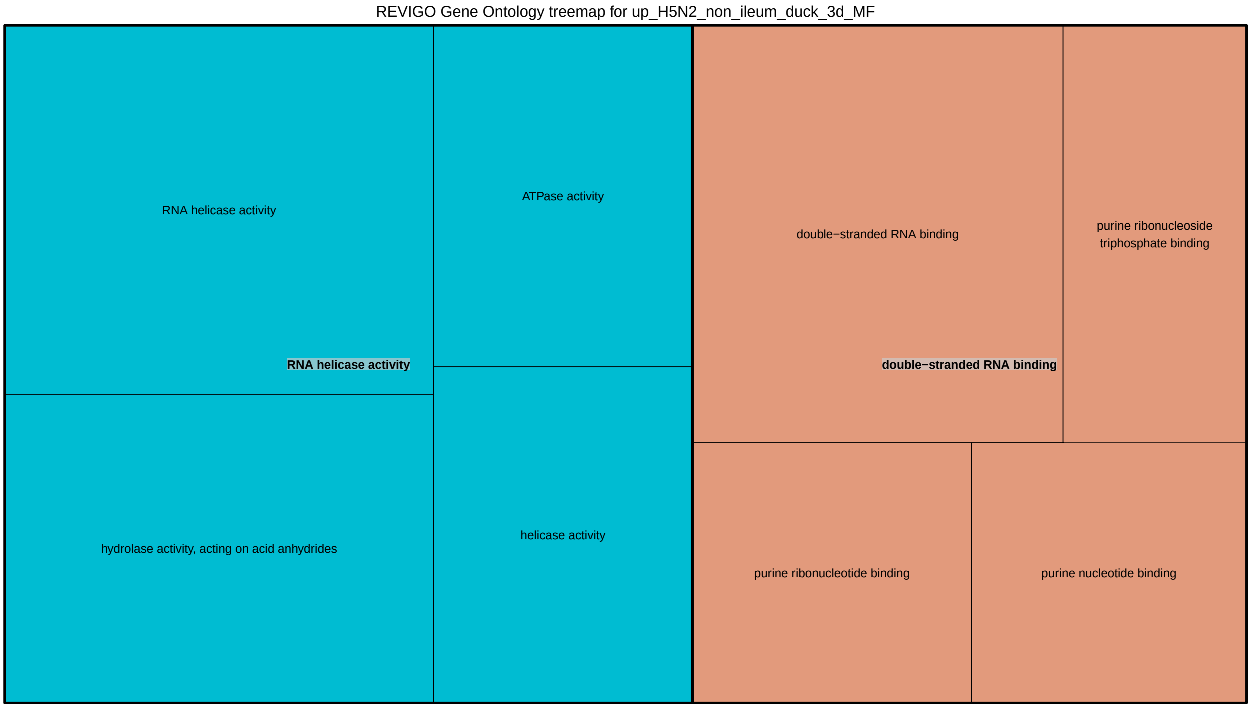

Figure S2.13: GO treemap for functionally enriched (P < 0.05) upregulated DEGs derived under the following experimental condition: ducks infected with H5N2 vs. mock-infected control measured in the ileum at 3 dpi. The boxes are grouped together based on the upper-hierarchy GO-term which is written in bold letters. Source = GO: Molecular functions
