# Supplementary material for: Comparative Investigation of Gene Regulatory Processes Underlying Avian Influenza Viruses in Chicken and Duck
Source: Biology (Basel). 2022 Jan 29;11(2):219. doi: 10.3390/biology11020219 (PMC8868632; doi:10.3390/biology11020219)
Supplement: Supplementary file 1 [file biology-11-00219-s001.zip › Supplementary_Figure_S4_master_regulators.pdf]

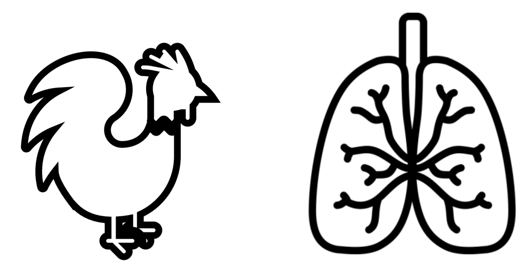

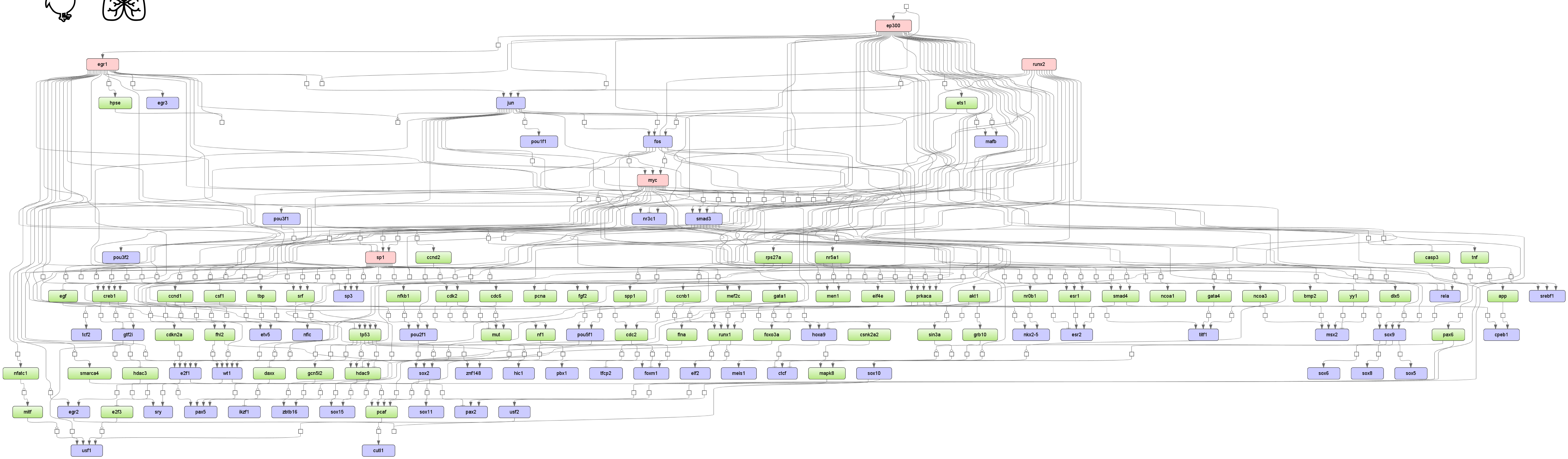

Figure S4.1: Scheme of the upstream regulatory network revealing the top five master regulators (red filled boxes) for the chicken lung after H5N1 infection (combining 1 and 3 dpi) based on the TF-TF cooperation results. The network was created and illustrated using the GeneXplain platform.

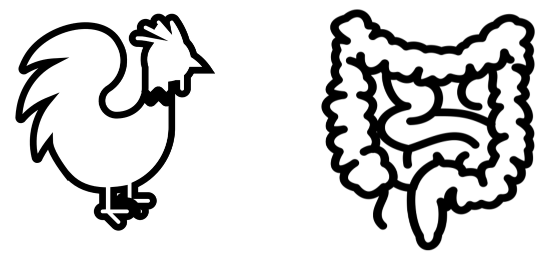

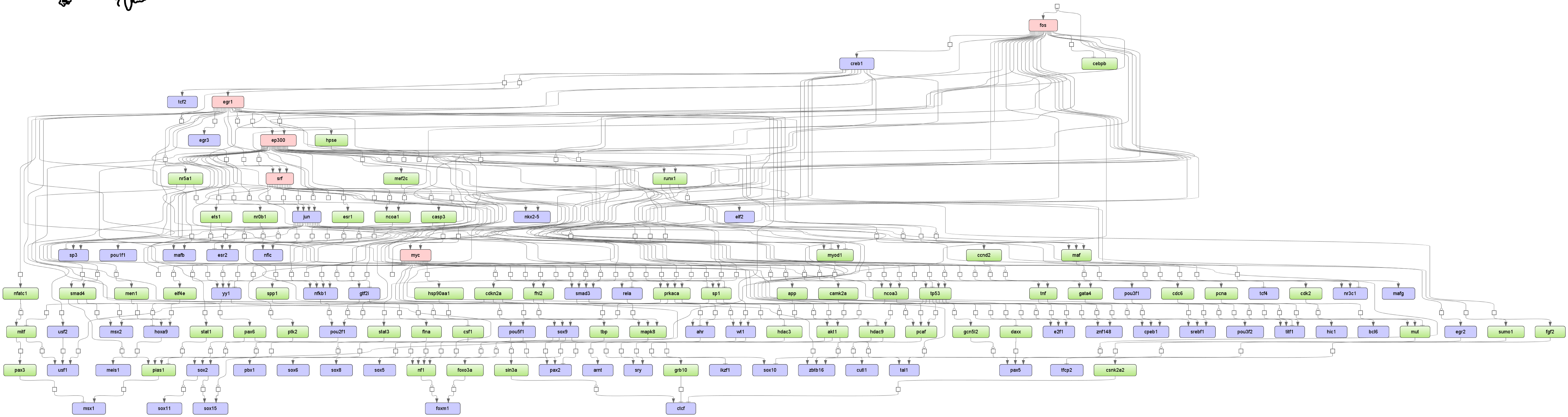

Figure S4.1: Scheme of the upstream regulatory network revealing the top five master regulators (red filled boxes) for the chicken ileum after H5N1 infection (combining 1 and 3 dpi) based on the TF-TF cooperation results. The network was created and illustrated using the GeneXplain platform.

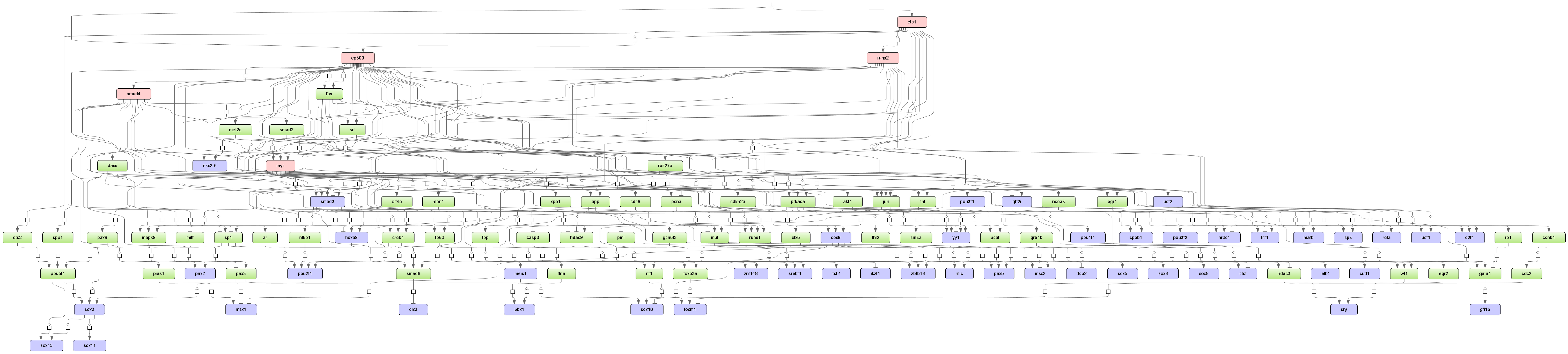

Figure S4.1: Scheme of the upstream regulatory network revealing the top five master regulators (red filled boxes) for the duck lung after H5N1 infection (combining 1 and 3 dpi) based on the TF-TF cooperation results. The network was created and illustrated using the GeneXplain platform.

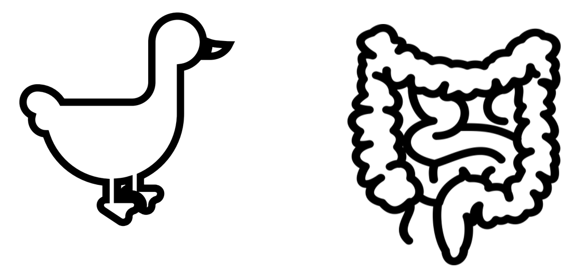

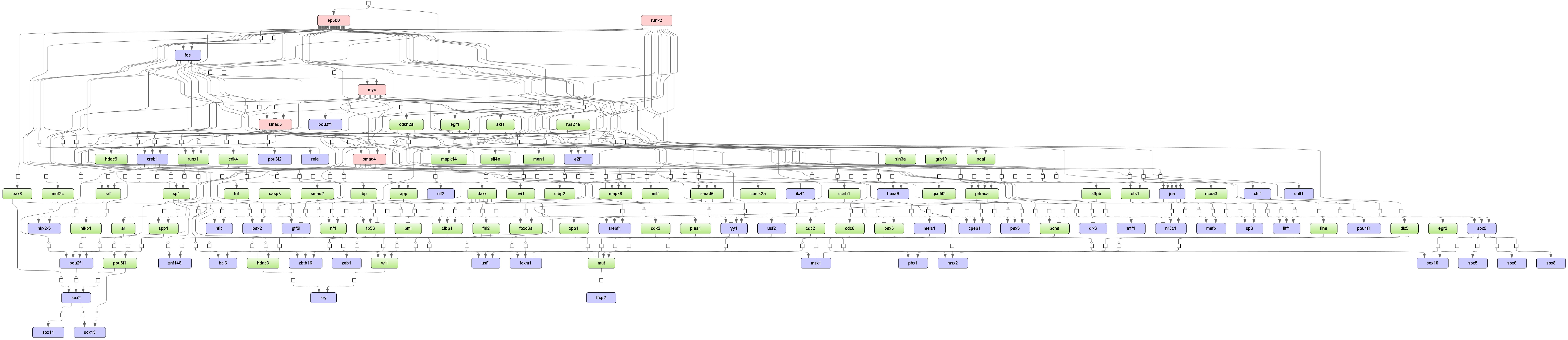

Figure S4.1: Scheme of the upstream regulatory network revealing the top five master regulators (red filled boxes) for the duck ileum after H5N1 infection (combining 1 and 3 dpi) based on the TF-TF cooperation results. The network was created and illustrated using the GeneXplain platform.
