# Supplementary figures and images for: Comparative Investigation of Gene Regulatory Processes Underlying Avian Influenza Viruses in Chicken and Duck
Source: Biology (Basel). 2022 Jan 29;11(2):219. doi: 10.3390/biology11020219 (PMC8868632; doi:10.3390/biology11020219)

A)

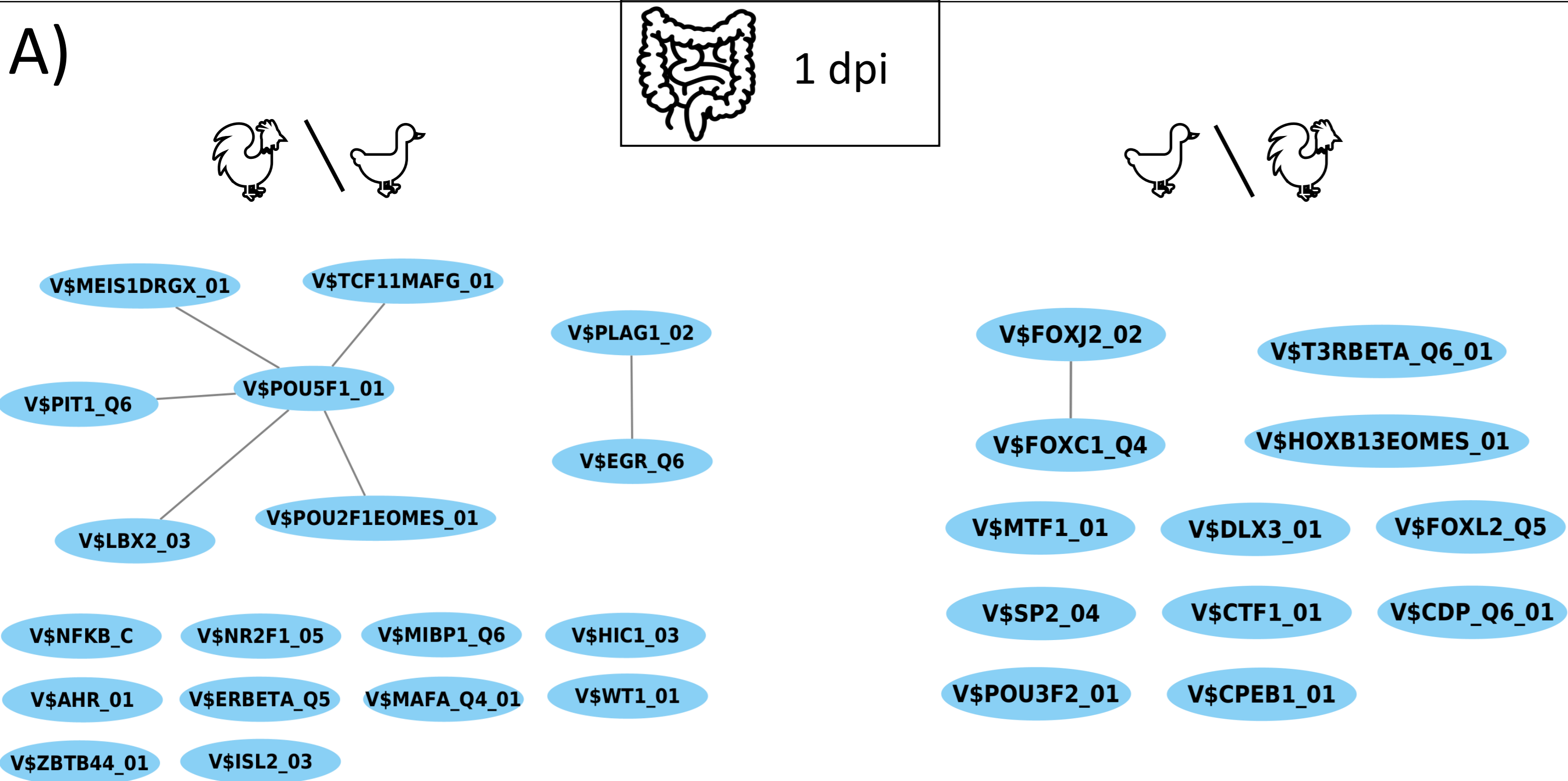

B)

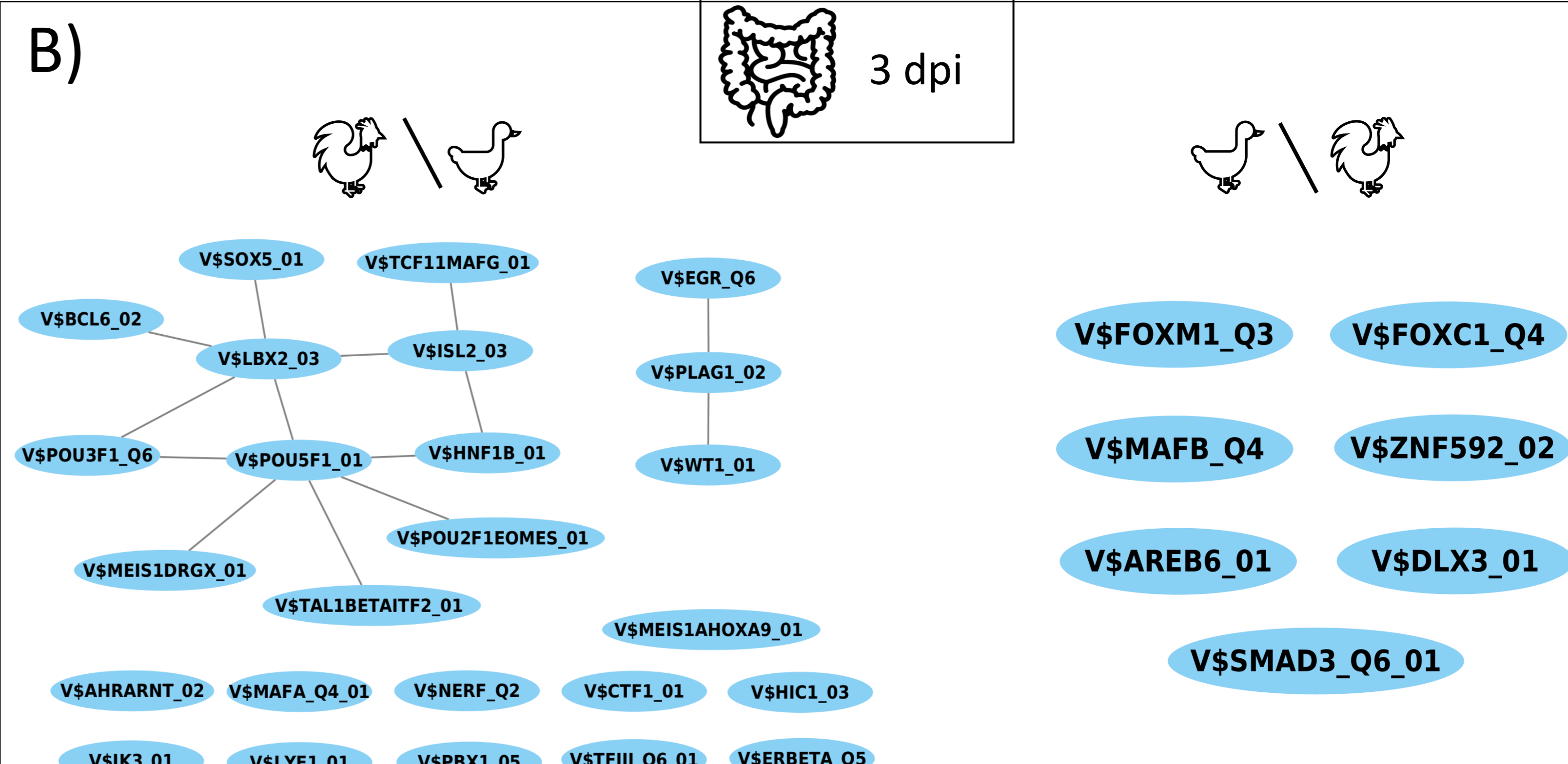

C)

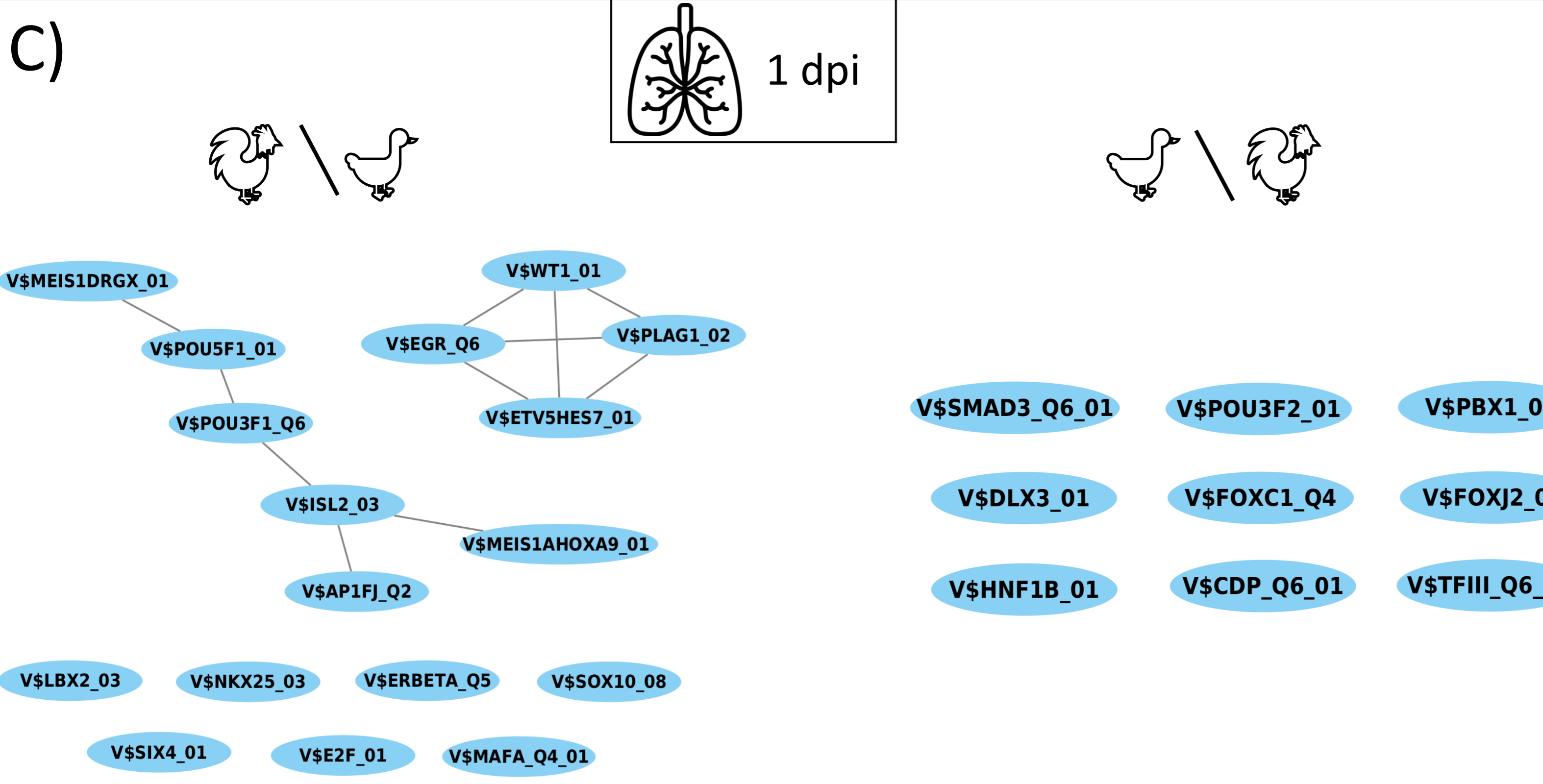

D)

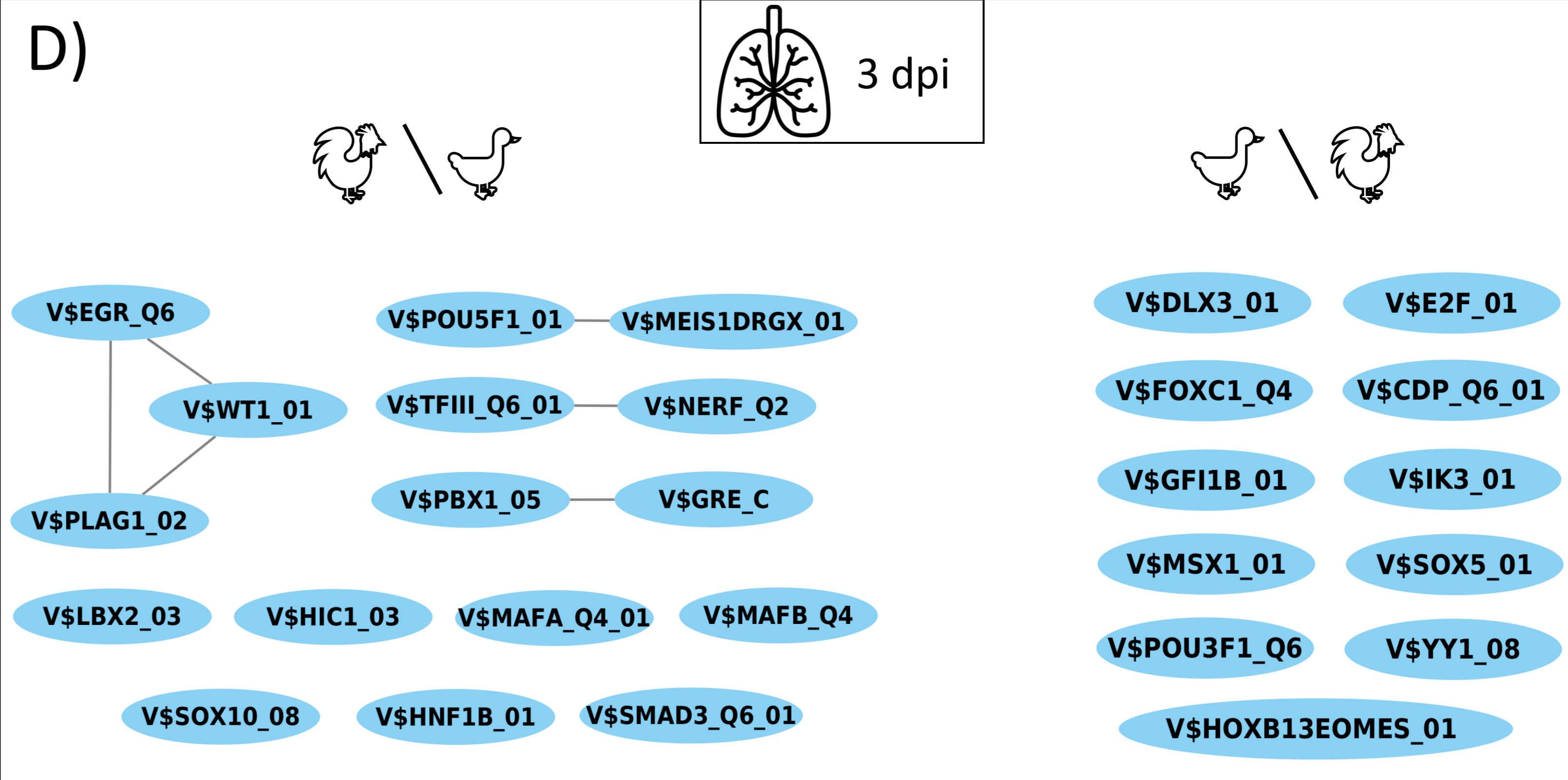

Supplement: Supplementary file 1 [file biology-11-00219-s001.zip › Supplementary_Figure_S3_Fig4.pdf]
